# Supplementary material for: The honesty behind tears: Situational, individual, and cultural influences on the perception of emotional tears as sincere
Source: PLoS One. 2025 Jul 16;20(7):e0324954. doi: 10.1371/journal.pone.0324954 (PMC12266444; doi:10.1371/journal.pone.0324954)
Supplement: S1 Note — (DOCX) [file pone.0324954.s001.docx]

**Supplementary Note S1**

**Preliminary Study: Re-Analysis of the CCT Project**

As a first step, we re-analyzed data collected by the CCT project (Zickfeld et al., 2021), focusing on perceived honesty. These analyses have not been published elsewhere and all data are openly available for a reanalysis. With the dataset, we are able to partly test H1 and H3, but importantly, the dataset had been originally intended for a different research question, and hence, our analyses were exploratory. Nevertheless, the dataset provided a first starting point as it includes ratings of perceived honesty provided by a large number of participants from 41 countries worldwide.

**Method**

***Participants***

The CCT project (Zickfeld et al., 2021) collected data from 7,007 individuals (4,474 women, 1,975 men, 45 other) between the age of 18 and 75 years (*M* = 28.08, *SD* = 10.89) across 41 countries, after exclusions. Recruitment details, including the number of individuals recruited in each country, are provided in Zickfeld et al. (2021; Table 1).

***Procedure***

The CCT employed a within-participant design, with each participant being presented with four targets, including always two non-tearful and two tearful targets (in random order). Targets were selected from a pool of 100 portraits taken from the Chicago Face Database (Ma et al., 2015) and the Bogazici database (Saribay et al., 2018) to represent male and female targets of five different ethnic groups (Asian, Black, Latinx, Middle East, White). The full pool consisted of 50 female and 50 male targets. Within the pool, 20 targets (10 male, 10 female) represented each ethnic group. Moreover, each of these pictures had a tearful version with digital tears added. Each target was accompanied by a short pre-tested situational description that varied in valence (positive, negative, or neutral) and social context (public or private). Positive and negative situations included highly emotional contexts such as reuniting or saying goodbye to a loved one. Neutral situations referred to everyday activities such as doing the laundry. For each target, it was randomly decided what target gender, ethnic group, the valence of the situation, and the type of social context participants were presented with. A detailed overview is provided in Zickfeld et al. (2021).

***Materials***

After each target, participants completed the same measures. For the current investigation, we focus on perceived honesty (how *honest* and *reliable* the target is perceived), perceived appropriateness (how *appropriate* the expression is perceived), and social support intentions (*I would be there if this person needed me*, *I would express how much I accept this person*, and *I would offer support to this person*). These measures were completed on a 7-point scale from 0 (*not at all*) to 6 (*very much*). A detailed overview of all measures is provided in Zickfeld et al. (2021) and Wróbel et al. (2022; Table 1).

**Results**

All analyses were conducted using R (R Project, 2023, version X.X.X). For main analyses we employed the following packages: *tidyverse* (Wickham et al., 2019; version 1.3.2), *lme4* (Bates et al., 2014; version 1.1-32), *emmeans* (Lenth, 2023, version 1.8.5), *sjPlot* (Lüdecke, 2023, version 2.8.14), *metafor* (Viechtbauer, 2010; version 3.8-1). Following Zickfeld et al. (2021) and Wróbel et al. (2022), we analyzed the data using multilevel models nesting individuals in country and target in ethnic group as random effects with random intercepts. In addition, we performed random-effects meta-analysis across countries. For all analyses, alpha level was set at 0.05.

We computed a perceived honesty score by averaging the two items. Internal reliabilities for each country separately, as well as descriptive statistics for perceived honesty by occurrence of tears is provided in Table S1. The three items on support intentions were also averaged to derive a total score (α = 0.87).

Testing the effect of occurrence of tears on perceived honesty, we observed that tearful targets were perceived as more honest compared to non-tearful targets, *d* = .28, 95% CI [.23, .33], *p* < 0.001. We observed significant heterogeneity across countries, *Q*(40) = 127.03, *p* < 0.001, *I2* = 70.73 [56.37, 83.76] (Figure 2a). All countries except for the United Arab Emirates showed positive effects, but these differed in magnitude with for example South Africa showing a small positive effect, *d* = 0.06 [-0.09, 0.21] and Norway showing a relatively stronger effect, *d* = 0.52 [0.37, 0.67].

**Supplementary Table S1.** Overview of perceived honesty ratings by occurrence of tears across the 41 countries

| Country | *r* | *No Tears* | | | *Tears* | | | *d* | 95% CI | |
| --- | --- | --- | --- | --- | --- | --- | --- | --- | --- | --- |
|  |  | *M* | *SD* | *n* | *M* | *SD* | *n* |  |  |  |
| Argentina | 0.78 | 3.27 | 1.32 | 236 | 3.51 | 1.37 | 240 | 0.18 | 0.00 | 0.36 |
| Australia | 0.85 | 3.35 | 1.22 | 146 | 3.71 | 1.19 | 146 | 0.30 | 0.07 | 0.53 |
| Austria | 0.74 | 3.64 | 1.07 | 327 | 3.89 | 1.04 | 331 | 0.24 | 0.09 | 0.40 |
| Bosnia & Herzegovina | 0.78 | 3.40 | 1.50 | 99 | 3.74 | 1.26 | 104 | 0.24 | -0.03 | 0.52 |
| Brazil | 0.62 | 3.29 | 1.42 | 175 | 3.39 | 1.26 | 188 | 0.07 | -0.13 | 0.28 |
| Canada | 0.82 | 3.33 | 1.22 | 388 | 3.53 | 1.25 | 389 | 0.16 | 0.02 | 0.30 |
| Chile | 0.74 | 2.99 | 1.43 | 131 | 3.52 | 1.36 | 128 | 0.38 | 0.13 | 0.62 |
| China | 0.65 | 2.95 | 1.18 | 374 | 3.27 | 1.25 | 382 | 0.27 | 0.12 | 0.41 |
| Colombia | 0.76 | 3.09 | 1.33 | 168 | 3.70 | 1.27 | 161 | 0.47 | 0.25 | 0.68 |
| Croatia | 0.77 | 3.41 | 1.19 | 247 | 3.61 | 1.14 | 242 | 0.16 | -0.01 | 0.34 |
| Finland | 0.80 | 3.50 | 1.20 | 455 | 3.95 | 1.15 | 456 | 0.39 | 0.26 | 0.52 |
| France | 0.79 | 3.15 | 1.37 | 1119 | 3.43 | 1.32 | 1115 | 0.21 | 0.12 | 0.29 |
| Germany | 0.77 | 3.63 | 1.21 | 533 | 4.05 | 1.08 | 539 | 0.37 | 0.25 | 0.49 |
| Greece | 0.78 | 3.18 | 1.29 | 110 | 3.75 | 1.29 | 107 | 0.44 | 0.17 | 0.71 |
| Hungary | 0.77 | 3.24 | 1.18 | 181 | 3.95 | 1.22 | 181 | 0.59 | 0.38 | 0.80 |
| India | 0.48 | 3.43 | 1.25 | 184 | 3.52 | 1.25 | 178 | 0.07 | -0.14 | 0.28 |
| Ireland | 0.80 | 3.39 | 1.11 | 157 | 3.58 | 1.27 | 156 | 0.16 | -0.06 | 0.38 |
| Israel | 0.69 | 3.18 | 1.28 | 704 | 3.67 | 1.26 | 706 | 0.39 | 0.28 | 0.49 |
| Japan | 0.81 | 2.84 | 1.11 | 329 | 3.42 | 1.19 | 326 | 0.50 | 0.34 | 0.65 |
| Malaysia | 0.71 | 3.00 | 1.16 | 154 | 3.28 | 1.14 | 150 | 0.24 | 0.02 | 0.47 |
| Mexico | 0.72 | 3.33 | 1.29 | 405 | 3.80 | 1.31 | 407 | 0.36 | 0.22 | 0.50 |
| Netherlands | 0.75 | 3.28 | 1.06 | 688 | 3.78 | 1.00 | 681 | 0.48 | 0.38 | 0.59 |
| New Zealand | 0.82 | 3.55 | 1.25 | 154 | 3.81 | 1.22 | 152 | 0.21 | -0.01 | 0.43 |
| Nigeria | 0.78 | 3.19 | 1.32 | 133 | 3.52 | 1.27 | 125 | 0.25 | 0.01 | 0.50 |
| Norway | 0.81 | 3.26 | 1.34 | 349 | 3.91 | 1.15 | 345 | 0.52 | 0.37 | 0.67 |
| Pakistan | 0.43 | 3.07 | 1.32 | 233 | 3.34 | 1.41 | 241 | 0.20 | 0.02 | 0.38 |
| Peru | 0.72 | 2.91 | 1.19 | 232 | 3.15 | 1.26 | 219 | 0.20 | 0.01 | 0.38 |
| Philippines | 0.68 | 3.14 | 1.26 | 191 | 3.41 | 1.32 | 190 | 0.21 | 0.00 | 0.41 |
| Poland | 0.71 | 2.87 | 1.27 | 147 | 3.10 | 1.27 | 148 | 0.18 | -0.04 | 0.41 |
| Portugal | 0.75 | 3.18 | 1.18 | 298 | 3.38 | 1.23 | 296 | 0.17 | 0.01 | 0.33 |
| Serbia | 0.75 | 3.38 | 1.15 | 247 | 3.76 | 1.14 | 241 | 0.32 | 0.15 | 0.50 |
| Singapore | 0.84 | 3.03 | 1.29 | 447 | 3.41 | 1.23 | 445 | 0.30 | 0.17 | 0.43 |
| Slovakia | 0.75 | 3.13 | 1.24 | 192 | 3.92 | 1.18 | 192 | 0.65 | 0.45 | 0.86 |
| South Africa | 0.70 | 3.46 | 1.32 | 341 | 3.55 | 1.43 | 365 | 0.06 | -0.09 | 0.21 |
| South Korea | 0.81 | 2.59 | 1.27 | 248 | 2.98 | 1.40 | 257 | 0.29 | 0.12 | 0.47 |
| Spain | 0.68 | 3.07 | 1.08 | 309 | 3.39 | 1.23 | 304 | 0.28 | 0.12 | 0.44 |
| Thailand | 0.73 | 2.97 | 1.14 | 217 | 3.26 | 1.15 | 214 | 0.25 | 0.06 | 0.44 |
| Turkey | 0.77 | 2.68 | 1.30 | 902 | 3.16 | 1.31 | 924 | 0.36 | 0.27 | 0.46 |
| United Arab Emirates | 0.79 | 3.71 | 1.10 | 144 | 3.35 | 1.19 | 144 | -0.31 | -0.54 | -0.08 |
| United Kingdom | 0.76 | 3.08 | 1.23 | 138 | 3.36 | 1.11 | 140 | 0.23 | 0.00 | 0.47 |
| United States | 0.83 | 3.12 | 1.55 | 203 | 3.38 | 1.59 | 196 | 0.16 | -0.03 | 0.36 |
|  |  |  |  |  |  |  |  |  |  |  |
| Total | 0.68 | 3.18 | 1.27 | 12364 | 3.54 | 1.27 | 12391 | 0.28 | 0.23 | 0.33 |

*Note.* *n* refers to the number of individual observations.

We tested whether this effect was moderated by situational valence and observed a statistically significant interaction (*t*(21560) = 20.47, *p* < 0.001). On average, tears increased perceived honesty for negative (non-tearful: *M* = 3.18, *SE* = 0.06; tearful: *M* = 3.74, *SE* = 0.06; *d* = 0.58 [0.53, 0.63]) and positive situations (non-tearful: *M* = 3.09, *SE* = 0.06; tearful: *M* = 3.64, *SE* = 0.06; *d* = 0.57 [0.53, 0.62]), but not for neutral situations (non-tearful: *M* = 3.27, *SE* = 0.06; tearful: *M* = 3.23, *SE* = 0.06; *d* = -0.04 [-0.09, 0.005]; Figure 2b). This effect was not moderated by perceived appropriateness (*F*(2,22042) = 2.71, *p* = 0.067; Figure S1). Perceptions of honesty increased with perceived appropriateness regardless of occurrence of tears or situational valence.


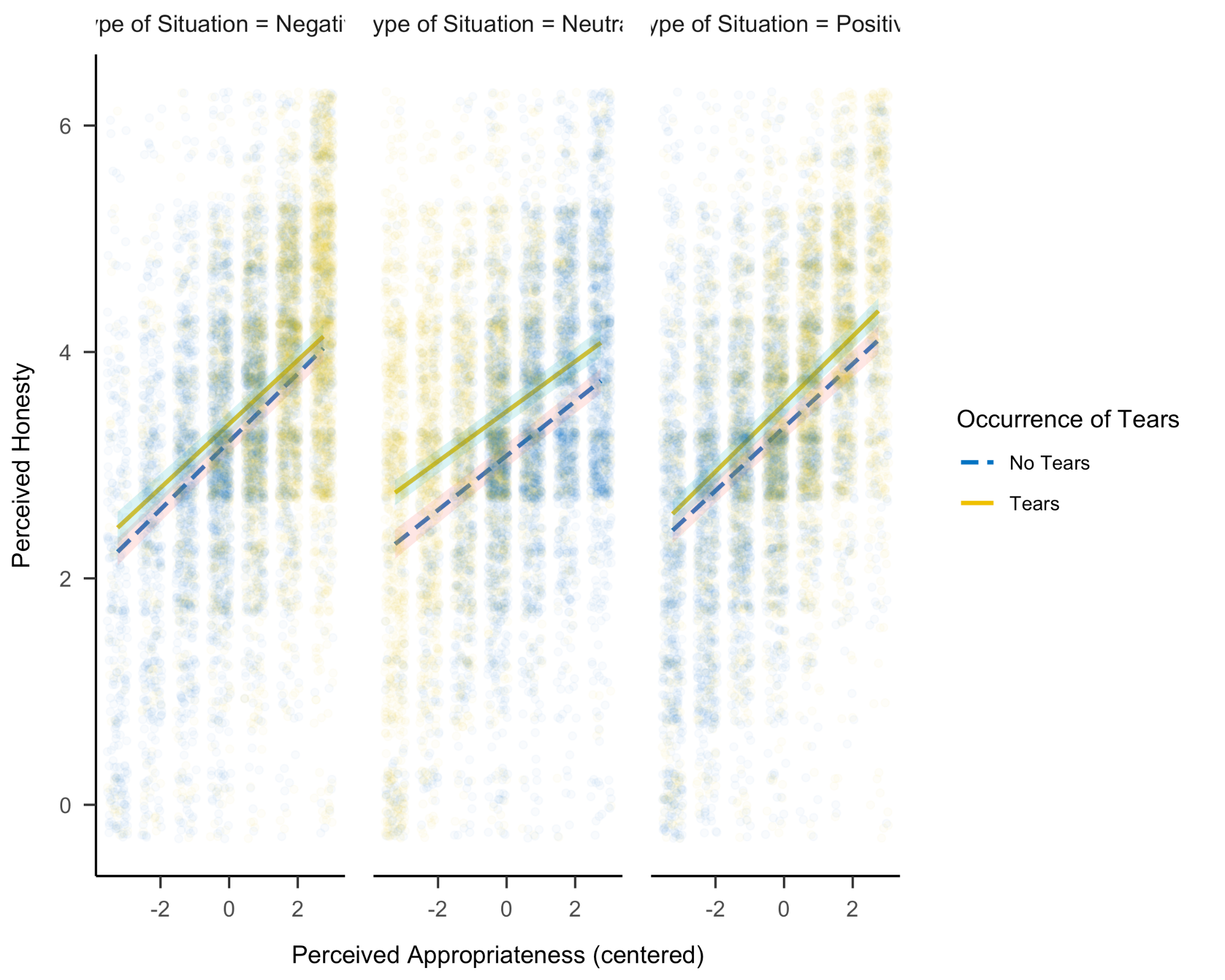


**Supplementary Figure S1.** Overview of the three-way interaction between occurrence of tears, type of situation, and perceived appropriateness on perceived honesty. Grey bands indicate 95% confidence intervals.

In addition, we found a significant interaction with target gender (*t*(21550) = -3.74, *p* < 0.001) with the effect of occurrence of tears on perceived honesty being slightly stronger for male targets (non-tearful: *M* = 3.13, *SE* = 0.06; tearful: *M* = 3.54, *SE* = 0.06, *d* = 0.43 [0.39, 0.46]) compared to female targets (non-tearful: *M* = 3.24, *SE* = 0.06; tearful: *M* = 3.55, *SE* = 0.06, *d* = 0.32 [0.28, 0.36]) due to lower levels of perceived honesty for non-tearful males.

Finally, we found that perceived honesty partially mediated the effect of occurrence of tears on support intentions (indirect effect: β = 0.06 [0.05, 0.07], Figure 2d).
