# Supplementary material for: The honesty behind tears: Situational, individual, and cultural influences on the perception of emotional tears as sincere
Source: PLoS One. 2025 Jul 16;20(7):e0324954. doi: 10.1371/journal.pone.0324954 (PMC12266444; doi:10.1371/journal.pone.0324954)
Supplement: S2 Note — (DOCX) [file pone.0324954.s002.docx]

**Supplementary Note S2**

**Country Selection**

We selected potential countries for inclusion in Study 1 based on two indices: 1.) effects of tears on perceived honesty as found in the CCT survey (Zickfeld et al., 2021; Figure 2a, Table S1) and 2.) general levels of trust based on the World Value Survey (Haerpfer et al., 2020) and the South African Social Attitudes Survey (https://hsrc.ac.za/special-projects/sasas/).

Originally, we selected seven countries and plotted their values of honesty perceptions and trust scores in Figure S2. We selected one country scoring high on honesty perceptions and trust (Norway), one country scoring average on honesty perceptions and high on trust (Canada), one country scoring average on honesty perceptions and low on trust (Poland), and one country scoring low on honesty perceptions and low on trust (South Africa).


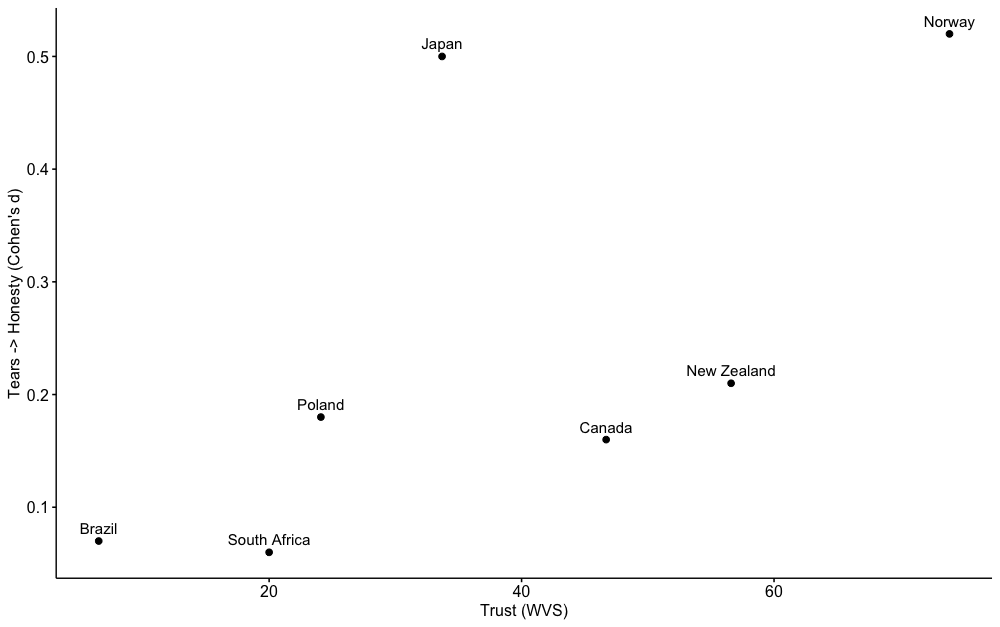


**Supplementary Figure S2.** Overview of country-level scores for honesty perceptions of tears (Zickfeld et al., 2021) and trust (World Value Survey; South African Social Attitudes Survey).
