# Supplementary material for: The honesty behind tears: Situational, individual, and cultural influences on the perception of emotional tears as sincere
Source: PLoS One. 2025 Jul 16;20(7):e0324954. doi: 10.1371/journal.pone.0324954 (PMC12266444; doi:10.1371/journal.pone.0324954)
Supplement: S3 Note — (DOCX) [file pone.0324954.s003.docx]

**Supplementary Note S3**

**Vignette Ratings**

We generated possible situations in which people would cry for manipulative reasons based on two main approaches. First, we reviewed 305 situations described by participants in a survey asked about contexts in which they or others would use emotional crying as a manipulative tactic (250 UK-based Prolific.com participants; 122 men, 125 women, 3 other, *M*_age_ = 40.58; see https://osf.io/wbyt2/). Second, we discussed potential situations with students. This resulted in a preliminary list of 55 potential situations. These differed on whether they included a partner, close other (e.g., family member), boss, colleague, or *other* as the target of crying. All authors then rated whether to include or exclude these situations for a rating study based on their potential of including emotional crying and featuring similar levels of morality. All situations for which at least two coders suggested inclusion were kept for the rating study. This included a total of 32 vignettes for the rating study, as summarized in Table S2. The majority of these included a partner (*n* = 9) or a close other (*n* = 9) as the target of manipulative crying, followed by a boss (*n* = 8), co-worker (*n* = 3), or other target (*n* = 3).

**Supplementary Table S2.** Overview of vignettes prepared for the rating study.

| **#** | **Vignette** | **Target** | **Selected** |
| --- | --- | --- | --- |
| 1 | X is trying to make their partner forgive them for cheating on them. | Partner |  |
| **2** | **X is trying to prevent their partner from breaking up with them.** | **Partner** | **x** |
| 3 | X is trying to make their partner spend holidays with their family. | Partner |  |
| **4** | **X is trying to make their partner stay at home instead of going out.** | **Partner** | **x** |
| 5 | X is trying to make their family member stop asking them difficult questions they do not want to answer. | Close Other |  |
| **6** | **X is trying to win an argument with their partner.** | **Partner** | **x** |
| 7 | X is trying to get their friend's attention. | Close Other |  |
| 8 | X is trying to make their partner help them with the kids. | Partner |  |
| 9 | X is trying to make their partner help them with household chores. | Partner |  |
| 10 | X is trying to make their family member apologize to them. | Close Other |  |
| 11 | X is trying to make their family member buy them an expensive gift. | Close Other |  |
| 12 | X is trying to make their family member pay off their debts. | Close Other |  |
| **13** | **X is trying to make their friend lend them some money.** | **Close Other** | **x** |
| 14 | X is trying to make their friend give them a ride. | Close Other |  |
| 15 | X is trying to make their friend attend an event they do not want to go to. | Close Other |  |
| **16** | **X is trying to make their partner move to another city with them.** | **Partner** | **x** |
| **17** | **X is trying to make their partner let them adopt a dog.** | **Partner** | **x** |
| 18 | X is trying to make their friend let them stay overnight at their place. | Close Other |  |
| 19 | X is trying to make their boss promote them. | Boss |  |
| 20 | X is trying to make their boss give them a raise. | Boss |  |
| 21 | X is trying to make their boss extend their contract. | Boss |  |
| 22 | X is trying to make their boss allow them to leave work early. | Boss |  |
| 23 | X is trying to make their boss give them a day off. | Boss |  |
| **24** | **X is trying to make their boss think they are overworked.** | **Boss** | **x** |
| 25 | X is trying to make their boss not give them a task they do not want do. | Boss |  |
| 26 | X is trying to make their boss turn a blind eye at their being late for work. | Boss |  |
| 27 | X is trying to get out of a difficult/unwanted task and make their colleague do their job. | Colleague |  |
| 28 | X is trying to get their colleague help them with their work. | Colleague |  |
| 29 | X is trying to make a co-worker change shifts with them. | Colleague |  |
| **30** | **X is trying to make a medical receptionist let them jump a waiting list to see a doctor.** | **Other** | **x** |
| **31** | **X is trying to make a passerby give them some money (for food, for a train home etc.)** | **Other** | **x** |
| **32** | **X is trying to make their professor give them an extension on an assignment.** | **Other** | **x** |

A total of 113 UK-based participants (55 men, 50 women, 4 other, 4 NA; *M*_age_ = 38.2, *SD*_age_ = 13.7) were recruited on Prolific.com. Each participant was asked to rate 10 vignettes randomly selected from the total pool of 32. Each vignette was rated on the three dimensions of manipulativeness (*How likely are people to use manipulation in this situation*?), tears/crying probability (*How likely are people to cry/shed tears in this situation?*), and morality (*How morally right or wrong do you think the person’s behavior in this situation is?*). Ratings were made on a 5-point scale from *very unlikely* or *very morally wrong* (1) to *very likely* or *very morally right* (5). An overview of ratings per vignette is provided in Figure S3. Based on the ratings, we selected 10 vignettes that scored high on manipulativeness (*Mdn* ≥ 3.5) and tears/crying probability (*Mdn* ≥ 3), and medium on morality (2 ≥ *Mdn* ≥ 3). For each vignette, we created a *neutral* version. The final selected vignettes and their neutral counterparts are presented in Table S3.

**
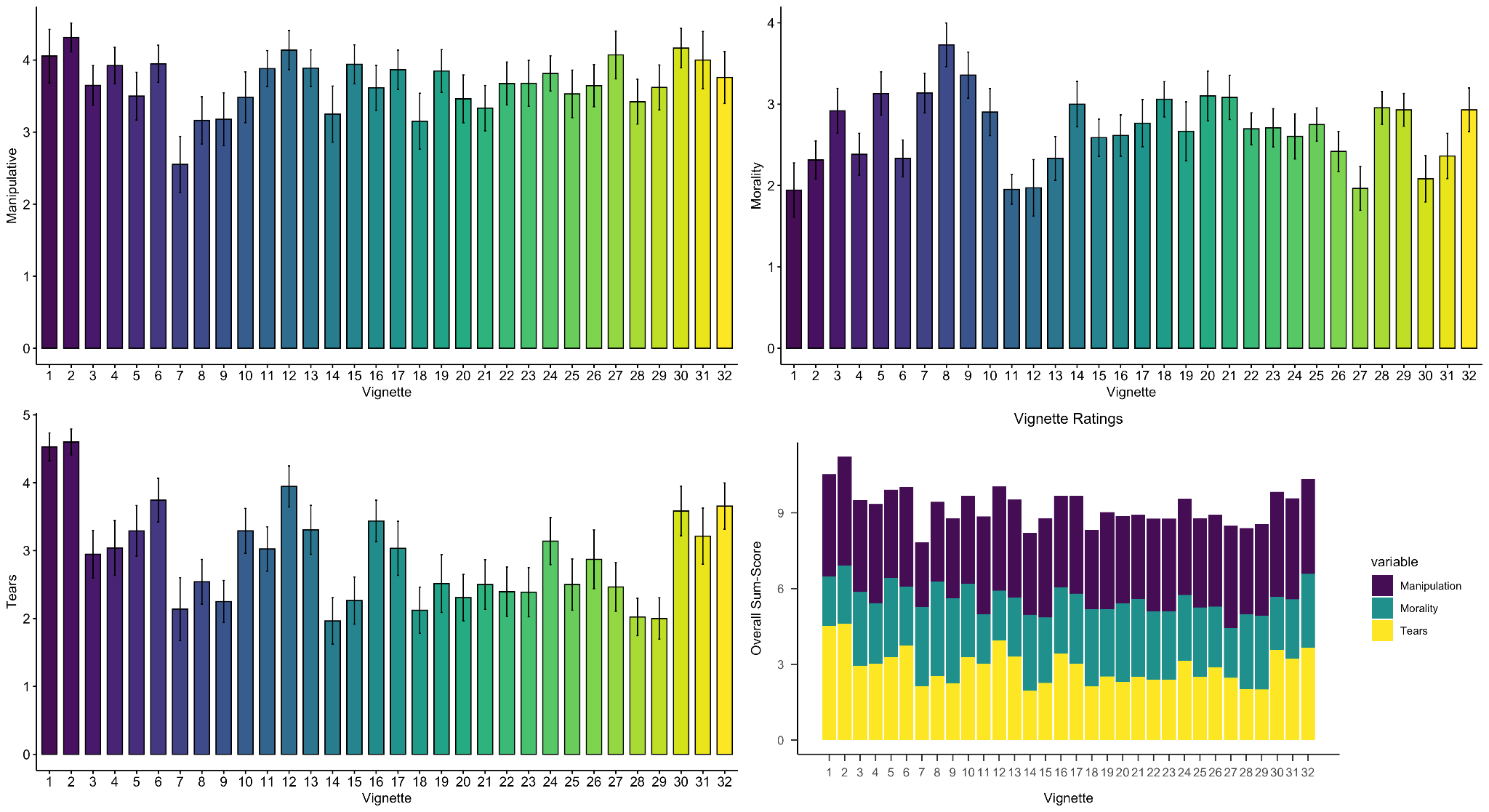
**

**Supplementary Figure S3.** Overview of ratings of manipulativeness, morality, the likelihood of including someone crying, and overall sum score of all ratings across all vignettes in the rating study. Error bars represent 95% confidence intervals.

**Supplementary Table S3.** Overview of final selected vignettes and their *neutral* comparisons.

| **Manipulative** | ***Neutral*** | ***Vignette No*** |
| --- | --- | --- |
| X was trying to prevent their partner from breaking up with them. | X's partner broke up with them | 7 |
| X was trying to make their partner stay at home instead of going out. | X did not feel like going out and wanted to stay home with their partner. | 8 |
| X was trying to win an argument with their partner. | X was arguing with their partner | 3 |
| X was trying to make their friend lend them some money. | X was talking with their friend about money problems they had | 5 |
| X was trying to make their partner move to another city with them. | X was talking with their partner about moving to another city. | 6 |
| X was trying to make their partner let them adopt a dog. | X wanted to adopt a dog and was telling their partner about it | 1 |
| X was trying to make their boss think they were overworked. | X felt overworked and was informing their boss about it. | 9 |
| X was trying to make a medical receptionist let them jump a waiting list to see a doctor. | X was waiting to see a doctor and was talking with a medical receptionist | 4 |
| X was trying to make a passerby give them some money (for food, for a train home etc.) | X wanted to get home and was asking a passerby for directions. | 2 |
| X was trying to make their professor give them an extension on an assignment. | X was talking with their professor about an assignment. | 10 |
