# Supplementary material for: The honesty behind tears: Situational, individual, and cultural influences on the perception of emotional tears as sincere
Source: PLoS One. 2025 Jul 16;20(7):e0324954. doi: 10.1371/journal.pone.0324954 (PMC12266444; doi:10.1371/journal.pone.0324954)
Supplement: S4 Note — (DOCX) [file pone.0324954.s004.docx]

**Supplementary Note S4**

**Study 1 Additional Analyses**

**Manipulation Check**

***Perceived Warmth.*** An overview of the main multilevel model for perceived warmth and high warmth, neutral, and low warmth pictures is presented in Supplementary Table S4. We followed-up by exploring the interaction with occurrence of tears as presented in Supplementary Table S5.

**Supplementary Table S4.** Overview of multilevel model for perceived warmth and face warmth

| *Predictors* | **Perceived Warmth** | | | | |
| --- | --- | --- | --- | --- | --- |
|  | *Estimates* | *std. Beta* | *CI* | *standardized CI* | *p* |
| (Intercept) | 4.19 | 0.31 | 4.06 – 4.31 | 0.23 – 0.39 | **<0.001** |
| Low-Warmth | -1.07 | -0.68 | -1.18 – -0.95 | -0.76 – -0.61 | **<0.001** |
| Neutral | -0.38 | -0.24 | -0.49 – -0.26 | -0.32 – -0.16 | **<0.001** |
| **Random Effects** | | | | | |
| σ^2^ | 1.70 | | | | |
| τ_00_ _ID:Country_ | 0.56 | | | | |
| τ_00_ _Country_ | 0.01 | | | | |
| ICC | 0.25 | | | | |
| N _ID_ | 942 | | | | |
| N _Country_ | 4 | | | | |
| Observations | 2782 | | | | |
| Marginal R^2^ / Conditional R^2^ | 0.080 / 0.308 | | | | |

*Note*. The reference level is high warmth for all comparisons.

**Supplementary Table S5.** Multilevel model of interaction between face dimension and occurrence of tears for perceived warmth.

| *Predictors* | **Perceived Warmth** | | |
| --- | --- | --- | --- |
|  | *Estimates* | *CI* | *p* |
| (Intercept) | 3.13 | 3.00 – 3.25 | **<0.001** |
| Face Dimension (Low-Warmth) | 0.69 | 0.58 – 0.81 | **<0.001** |
| Face Dimension (Neutral) | 1.07 | 0.95 – 1.18 | **<0.001** |
| Occurrence of Tears | 0.45 | 0.31 – 0.59 | **<0.001** |
| Face Dimension (Low-Warmth) × OoT | -0.45 | -0.61 – -0.28 | **<0.001** |
| Face Dimension (Neutral) × OoT | -0.73 | -0.89 – -0.57 | **<0.001** |
| **Random Effects** | | | |
| σ^2^ | 1.61 | | |
| τ_00_ _ID:Country_ | 0.65 | | |
| τ_00_ _Country_ | 0.01 | | |
| ICC | 0.29 | | |
| N _ID_ | 1891 | | |
| N _Country_ | 4 | | |
| Observations | 5581 | | |
| Marginal R^2^ / Conditional R^2^ | 0.046 / 0.323 | | |

*Note.* OoT = Occurrence of Tears. For face dimension reference category is high warmth. Occurrence of tears (-.5 = no tears, .5 = tears).

***Perceived Competence*.** For competence, we observed that neutral faces (*M* = 4.25, *SE* = .14) were perceived as more competent than low-competence faces (*M* = 4.05, *SE* = .14, *d* = .14 [.07, .22], *p* = < .001). However, we did not find evidence that high-competence faces (*M* = 4.12, *SE* = .14) were perceived as significantly more competent than low-competence (*d* = .05 [-.02, .12], *p* = .181) or neutral faces (*d* = -.09 [-.17, -.02], *p* = 0.011; Supplementary Table S6). Neutral faces were actually perceived as significantly more competent than high-competence faces. Therefore, our manipulation of competence failed. Note, that we did not register any main analyses focusing on this factor, but we will also refrain from performing any explorative analyses including this factor.

**Supplementary Table S6.** Multilevel model of face dimensions (low competence, neutral, high competence) for perceived competence.

| *Predictors* | **Perceived Competence** | | | | |
| --- | --- | --- | --- | --- | --- |
|  | *Estimates* | *std. Beta* | *CI* | *standardized CI* | *p* |
| (Intercept) | 4.12 | -0.01 | 3.83 – 4.41 | -0.22 – 0.21 | **<0.001** |
| Face Dimension (Low Competence) | -0.07 | -0.05 | -0.17 – 0.03 | -0.12 – 0.02 | 0.181 |
| Face Dimension (Neutral) | 0.13 | 0.09 | 0.03 – 0.23 | 0.02 – 0.17 | **0.011** |
| **Random Effects** | | | | | |
| σ^2^ | 1.19 | | | | |
| τ_00_ _ID:Country_ | 0.61 | | | | |
| τ_00_ _Country_ | 0.08 | | | | |
| ICC | 0.37 | | | | |
| N _ID_ | 941 | | | | |
| N _Country_ | 4 | | | | |
| Observations | 2774 | | | | |
| Marginal R^2^ / Conditional R^2^ | 0.004 / 0.368 | | | | |

*Note.* The reference category is high competence.

**Analysis with Registered Manipulativeness Measure**

***Perceived Manipulativeness*.** We tested whether targets depicted in manipulative contexts were perceived as more manipulative than targets depicted in non-manipulative contexts. We observed a significant difference for the index (*d* = .35 [.28, .42], *p* < .001). However, as this index showed a considerably low correlation across countries (Table 2), we tested the individual items *manipulative* and *knows how to behave to get what they want* separately and found a stronger effect for the *manipulative* item (*d* = .42 [.34, .49]) compared to the other item (*d* = .16 [.10, .22]). Hence, against our preregistration, we focused only on the *manipulative* item. We further explored the interaction between occurrence of tears and situational context for the perceived manipulativeness single item measure as depicted in Supplementary Table S7.

**Supplementary Table S7.** Multilevel model for interaction between occurrence of tears and situational context for perceived manipulativeness.

| *Predictors* | **Perceived Manipulativeness (Single Item)** | | | | |
| --- | --- | --- | --- | --- | --- |
|  | *Estimates* | *std. Beta* | *CI* | *standardized CI* | *p* |
| (Intercept) | 3.40 | -0.27 | 3.27 – 3.54 | -0.34 – -0.19 | **<0.001** |
| Situational Context | 0.59 | 0.34 | 0.44 – 0.73 | 0.26 – 0.42 | **<0.001** |
| Occurrence of Tears | 0.18 | 0.10 | 0.07 – 0.29 | 0.04 – 0.17 | **0.002** |
| Situational Context ×  Occurrence of Tears | 0.28 | 0.16 | 0.14 – 0.41 | 0.08 – 0.23 | **<0.001** |
| **Random Effects** | | | | | |
| σ^2^ | 2.33 | | | | |
| τ_00_ _ID:Country_ | 0.49 | | | | |
| τ_00_ _vgn_ | 0.04 | | | | |
| τ_00_ _Country_ | 0.01 | | | | |
| ICC | 0.19 | | | | |
| N _ID_ | 1893 | | | | |
| N _Country_ | 4 | | | | |
| N _vgn_ | 60 | | | | |
| Observations | 9296 | | | | |
| Marginal R^2^ / Conditional R^2^ | 0.054 / 0.232 | | | | |

*Note.* Occurrence of tears (-.5 = no tears, .5 = tears), situational context (-.5 = non-manipulative, .5 = manipulative).

**Detailed Mediation Models (H2)**

**Supplementary Table S8.** Multilevel model of occurrence of tears, perceived expression authenticity and perceived manipulativeness on perceived honesty (for dimensions of low and high warmth only).

| *Predictors* | **Perceived Honesty** | | | | |
| --- | --- | --- | --- | --- | --- |
|  | *Estimates* | *std. Beta* | *CI* | *standardized CI* | *p* |
| (Intercept) | 3.88 | -0.04 | 3.80 – 3.97 | -0.09 – 0.02 | **<0.001** |
| Occurrence of Tears | 0.12 | 0.08 | 0.04 – 0.19 | 0.03 – 0.12 | **0.002** |
| Perceived Manipulativeness | -0.19 | -0.21 | -0.21 – -0.17 | -0.23 – -0.19 | **<0.001** |
| Perceived Expression Authenticity | 0.60 | 0.66 | 0.58 – 0.62 | 0.64 – 0.68 | **<0.001** |
| **Random Effects** | | | | | |
| σ^2^ | 0.81 | | | | |
| τ_00_ _ID:Country_ | 0.25 | | | | |
| τ_00_ _pic_id_ | 0.00 | | | | |
| τ_00_ _Country_ | 0.00 | | | | |
| ICC | 0.24 | | | | |
| N _ID_ | 1883 | | | | |
| N _Country_ | 4 | | | | |
| N _pic_id_ | 30 | | | | |
| Observations | 3720 | | | | |
| Marginal R^2^ / Conditional R^2^ | 0.549 / 0.657 | | | | |

*Note.* Occurrence of tears (-0.5 no tears, 0.5 tears). All continuous predictors are mean-centered.

**Effect on Support Intentions (H3)**

**Supplementary Table S9.** Multilevel model of occurrence of tears, face warmth, and their interaction on support intentions.

| *Predictors* | **Support Intentions** | | | | |
| --- | --- | --- | --- | --- | --- |
|  | *Estimates* | *std. Beta* | *CI* | *standardized CI* | *p* |
| (Intercept) | 3.54 | -0.19 | 3.30 – 3.79 | -0.33 – -0.05 | **<0.001** |
| Occurrence of Tears | 0.33 | 0.20 | 0.18 – 0.49 | 0.11 – 0.28 | **<0.001** |
| Face Warmth | 0.55 | 0.32 | 0.43 – 0.68 | 0.25 – 0.40 | **<0.001** |
| Occurrence of Tears x Face Warmth | -0.49 | -0.29 | -0.66 – -0.31 | -0.39 – -0.18 | **<0.001** |
| **Random Effects** | | | | | |
| σ^2^ | 1.86 | | | | |
| τ_00_ _ID:Country_ | 0.93 | | | | |
| τ_00_ _pic_id_ | 0.04 | | | | |
| τ_00_ _Country_ | 0.05 | | | | |
| ICC | 0.35 | | | | |
| N _ID_ | 1883 | | | | |
| N _Country_ | 4 | | | | |
| N _pic_id_ | 30 | | | | |
| Observations | 3724 | | | | |
| Marginal R^2^ / Conditional R^2^ | 0.014 / 0.363 | | | | |

*Note.* Occurrence of tears (-.5 = no tears, .5 = tears); face warmth (-.5 = low warmth, .5 = high warmth).

**Moderation by Dark Triad (H4)**

**Supplementary Table S10.** Multilevel model of occurrence of tears, situational context, dark triad measures, and their interactions for perceived honesty.

|  | **Perceived Honesty** | | | | | |
| --- | --- | --- | --- | --- | --- | --- |
| *Predictors* | *Estimates* | *std. Beta* | *CI* | *standardized CI* | *p* | *std. p* |
| (Intercept) | 4.27 | 0.20 | 4.04 – 4.49 | 0.06 – 0.35 | **<0.001** | **0.007** |
| Occurrence of Tears | 0.00 | 0.00 | -0.10 – 0.10 | -0.06 – 0.07 | 0.944 | 0.945 |
| Situational Context | -0.52 | -0.34 | -0.60 – -0.44 | -0.39 – -0.29 | **<0.001** | **<0.001** |
| Psychopathy | 0.02 | 0.01 | -0.05 – 0.09 | -0.04 – 0.07 | 0.613 | 0.613 |
| Narcissism | 0.04 | 0.04 | -0.02 – 0.10 | -0.02 – 0.09 | 0.182 | 0.182 |
| Machiavellianism | 0.04 | 0.04 | -0.03 – 0.12 | -0.03 – 0.10 | 0.282 | 0.282 |
| Occurrence of Tears × Situational Context | -0.14 | -0.09 | -0.25 – -0.02 | -0.16 – -0.02 | **0.018** | **0.018** |
| Occurrence of Tears ×  Psychopathy | -0.09 | -0.07 | -0.19 – 0.01 | -0.15 – 0.01 | 0.090 | 0.090 |
| Situational Context ×  Psychopathy | 0.06 | 0.05 | -0.02 – 0.14 | -0.02 – 0.11 | 0.163 | 0.163 |
| Occurrence of Tears ×  Machiavellianism | -0.03 | -0.03 | -0.14 – 0.07 | -0.12 – 0.06 | 0.518 | 0.518 |
| Situational Context ×  Machiavellianism | -0.03 | -0.03 | -0.12 – 0.05 | -0.10 – 0.05 | 0.463 | 0.463 |
| Occurrence of Tears ×  Narcissism | 0.01 | 0.01 | -0.07 – 0.09 | -0.07 – 0.09 | 0.825 | 0.825 |
| Situational Context ×  Narcissism | 0.04 | 0.04 | -0.03 – 0.11 | -0.03 – 0.10 | 0.260 | 0.260 |
| (Occurrence of Tears × Situational Context) × Psychopathy | -0.02 | -0.01 | -0.13 – 0.10 | -0.11 – 0.08 | 0.773 | 0.773 |
| (Occurrence of Tears × Situational Context) ×  Machiavellianism | 0.09 | 0.08 | -0.03 – 0.21 | -0.03 – 0.18 | 0.151 | 0.151 |
| (Occurrence of Tears × Situational Context) × Narcissism | 0.00 | 0.00 | -0.09 – 0.10 | -0.09 – 0.09 | 0.953 | 0.953 |
| **Random Effects** | | | | | | |
| σ^2^ | 1.78 | | | | | |
| τ_00_ _ID:Country_ | 0.45 | | | | | |
| τ_00_ _pic_id_ | 0.04 | | | | | |
| τ_00_ _Country_ | 0.04 | | | | | |
| ICC | 0.23 | | | | | |
| N _ID_ | 1888 | | | | | |
| N _Country_ | 4 | | | | | |
| N _pic_id_ | 30 | | | | | |
| Observations | 9280 | | | | | |
| Marginal R^2^ / Conditional R^2^ | 0.046 / 0.266 | | | | | |

*Note.* Occurrence of tears (-.5 = no tears, .5 = tears); situational context (-.5 = non-manipulative, .5 = manipulative). Continuous predictors are mean-centered.

**Supplementary Table S11.** Multilevel models for each dark triad separately, occurrence of tears, situational context and interactions for perceived honesty.

| *Predictors* | **Perceived Honesty** | | | | | **Perceived Honesty** | | | | | | **Perceived Honesty** | | | | | |
| --- | --- | --- | --- | --- | --- | --- | --- | --- | --- | --- | --- | --- | --- | --- | --- | --- | --- |
|  | *Estimates* | *std. Beta* | *CI* | *standardized CI* | *p* | *Estimates* | *std. Beta* | *CI* | *standardized CI* | *p* | *std. p* | *Estimates* | *std. Beta* | *CI* | *standardized CI* | *p* | *std. p* |
| (Intercept) | 4.27 | 0.20 | 4.05 – 4.49 | 0.06 – 0.34 | **<0.001** | 4.27 | 0.20 | 4.04 – 4.50 | 0.05 – 0.35 | **<0.001** | **0.007** | 4.27 | 0.20 | 4.06 – 4.48 | 0.06 – 0.34 | **<0.001** | **0.004** |
| Occurrence of Tears | 0.01 | 0.00 | -0.10 – 0.11 | -0.06 – 0.07 | 0.920 | 0.00 | 0.00 | -0.10 – 0.10 | -0.06 – 0.07 | 0.948 | 0.949 | 0.00 | 0.00 | -0.10 – 0.10 | -0.06 – 0.07 | 0.944 | 0.944 |
| Situational Context | -0.52 | -0.34 | -0.60 – -0.44 | -0.39 – -0.29 | **<0.001** | -0.52 | -0.34 | -0.60 – -0.44 | -0.39 – -0.28 | **<0.001** | **<0.001** | -0.52 | -0.34 | -0.60 – -0.44 | -0.39 – -0.28 | **<0.001** | **<0.001** |
| OoT * SC | -0.14 | -0.09 | -0.26 – -0.02 | -0.17 – -0.02 | **0.017** | -0.14 | -0.09 | -0.26 – -0.02 | -0.16 – -0.02 | **0.018** | **0.018** | -0.14 | -0.09 | -0.26 – -0.03 | -0.17 – -0.02 | **0.016** | **0.016** |
| Psychopathy | 0.06 | 0.05 | 0.01 – 0.12 | 0.00 – 0.10 | **0.031** |  |  |  |  |  |  |  |  |  |  |  |  |
| OoT * Psychopathy | -0.10 | -0.08 | -0.19 – -0.02 | -0.15 – -0.02 | **0.012** |  |  |  |  |  |  |  |  |  |  |  |  |
| SC * Psychopathy | 0.05 | 0.04 | -0.01 – 0.12 | -0.01 – 0.10 | 0.109 |  |  |  |  |  |  |  |  |  |  |  |  |
| OoT * SC * Psychopathy | 0.05 | 0.04 | -0.05 – 0.14 | -0.04 – 0.11 | 0.336 |  |  |  |  |  |  |  |  |  |  |  |  |
| Narcisissm |  |  |  |  |  | 0.07 | 0.06 | 0.02 – 0.12 | 0.02 – 0.11 | **0.008** | **0.008** |  |  |  |  |  |  |
| OoT * Narcissism |  |  |  |  |  | -0.04 | -0.03 | -0.11 – 0.03 | -0.10 – 0.03 | 0.309 | 0.309 |  |  |  |  |  |  |
| SC × Narcisissm |  |  |  |  |  | 0.04 | 0.04 | -0.02 – 0.10 | -0.02 – 0.09 | 0.171 | 0.171 |  |  |  |  |  |  |
| OoT * SC * Narcissism |  |  |  |  |  | 0.05 | 0.04 | -0.03 – 0.13 | -0.03 – 0.12 | 0.267 | 0.267 |  |  |  |  |  |  |
| Machiavellianism |  |  |  |  |  |  |  |  |  |  |  | 0.08 | 0.06 | 0.02 – 0.13 | 0.02 – 0.11 | **0.006** | **0.006** |
| OoT ×  Machiavellianism |  |  |  |  |  |  |  |  |  |  |  | -0.08 | -0.07 | -0.15 – -0.00 | -0.13 – -0.00 | **0.048** | **0.048** |
| SC ×  Machiavellianism |  |  |  |  |  |  |  |  |  |  |  | 0.02 | 0.02 | -0.04 – 0.08 | -0.03 – 0.07 | 0.462 | 0.462 |
| OoT × SC ×  Machiavellianism |  |  |  |  |  |  |  |  |  |  |  | 0.08 | 0.07 | -0.00 – 0.17 | -0.00 – 0.15 | 0.061 | 0.061 |
| **Random Effects** | | | | | | | | | | | | | | | | | |
| σ^2^ | 1.78 | | | | | 1.78 | | | | | | 1.78 | | | | | |
| τ_00_ | 0.46 _ID:Country_ | | | | | 0.46 _ID:Country_ | | | | | | 0.46 _ID:Country_ | | | | | |
|  | 0.04 _pic_id_ | | | | | 0.04 _pic_id_ | | | | | | 0.04 _pic_id_ | | | | | |
|  | 0.04 _Country_ | | | | | 0.04 _Country_ | | | | | | 0.04 _Country_ | | | | | |
| ICC | 0.23 | | | | | 0.23 | | | | | | 0.23 | | | | | |
| N | 1888 _ID_ | | | | | 1889 _ID_ | | | | | | 1889 _ID_ | | | | | |
|  | 4 _Country_ | | | | | 4 _Country_ | | | | | | 4 _Country_ | | | | | |
|  | 30 _pic_id_ | | | | | 30 _pic_id_ | | | | | | 30 _pic_id_ | | | | | |
| Observations | 9280 | | | | | 9285 | | | | | | 9285 | | | | | |
| Marginal R^2^ / Conditional R^2^ | 0.041 / 0.263 | | | | | 0.044 / 0.265 | | | | | | 0.042 / 0.264 | | | | | |

*Note.* OoT = Occurrence of Tears, SC = Situational Context. Occurrence of tears (-.5 = no tears, .5 = tears); situational context (-.5 = non-manipulative, .5 = manipulative). Continuous predictors are mean-centered.

**Supplementary Table S12.** Multilevel models for each dark triad separately, occurrence of tears, situational context and interactions for perceived manipulativeness.

| *Predictors* | **Perceived Target Manipulativeness** | | | | | | **Perceived Target Manipulativeness** | | | | | **Perceived Target Manipulativeness** | | | | | | |
| --- | --- | --- | --- | --- | --- | --- | --- | --- | --- | --- | --- | --- | --- | --- | --- | --- | --- | --- |
|  | *Estimates* | *std. Beta* | *CI* | *standardized CI* | *p* | *std. p* | *Estimates* | *std. Beta* | *CI* | *standardized CI* | *p* | *Estimates* | *std. Beta* | *CI* | *standardized CI* | *p* | *std. p* |  |
| (Intercept) | 3.39 | -0.27 | 3.25 – 3.53 | -0.35 – -0.19 | **<0.001** | **<0.001** | 3.39 | -0.27 | 3.28 – 3.51 | -0.34 – -0.20 | **<0.001** | 3.39 | -0.27 | 3.28 – 3.50 | -0.33 – -0.21 | **<0.001** | **<0.001** |  |
| Occurrence of Tears | 0.18 | 0.10 | 0.07 – 0.29 | 0.04 – 0.16 | **0.002** | **0.002** | 0.17 | 0.10 | 0.06 – 0.28 | 0.04 – 0.16 | **0.002** | 0.17 | 0.10 | 0.06 – 0.28 | 0.03 – 0.16 | **0.002** | **0.002** |  |
| Situational Context | 0.61 | 0.35 | 0.52 – 0.71 | 0.30 – 0.41 | **<0.001** | **<0.001** | 0.61 | 0.35 | 0.51 – 0.70 | 0.30 – 0.40 | **<0.001** | 0.61 | 0.35 | 0.52 – 0.70 | 0.30 – 0.40 | **<0.001** | **<0.001** |  |
| OoT * SC | 0.27 | 0.16 | 0.14 – 0.40 | 0.08 – 0.23 | **<0.001** | **<0.001** | 0.28 | 0.16 | 0.15 – 0.41 | 0.09 – 0.24 | **<0.001** | 0.28 | 0.16 | 0.15 – 0.41 | 0.09 – 0.24 | **<0.001** | **<0.001** |  |
| Psychopathy | 0.23 | 0.17 | 0.17 – 0.30 | 0.12 – 0.21 | **<0.001** | **<0.001** |  |  |  |  |  |  |  |  |  |  |  |  |
| OoT * Psychopathy | -0.04 | -0.03 | -0.13 – 0.05 | -0.09 – 0.04 | 0.418 | 0.418 |  |  |  |  |  |  |  |  |  |  |  |  |
| SC * Psychopathy | -0.07 | -0.05 | -0.14 – 0.01 | -0.10 – 0.01 | 0.083 | 0.083 |  |  |  |  |  |  |  |  |  |  |  |  |
| OoT * SC * Psychopathy | 0.00 | 0.00 | -0.10 – 0.11 | -0.07 – 0.08 | 0.973 | 0.973 |  |  |  |  |  |  |  |  |  |  |  |  |
| Narcisissm |  |  |  |  |  |  | 0.19 | 0.16 | 0.14 – 0.25 | 0.12 – 0.21 | **<0.001** |  |  |  |  |  |  |  |
| OoT * Narcissism |  |  |  |  |  |  | -0.03 | -0.02 | -0.10 – 0.05 | -0.08 – 0.04 | 0.504 |  |  |  |  |  |  |  |
| SC ×  Narcisissm |  |  |  |  |  |  | -0.07 | -0.06 | -0.14 – -0.01 | -0.11 – -0.01 | **0.029** |  |  |  |  |  |  |  |
| OoT * SC * Narcissism |  |  |  |  |  |  | 0.02 | 0.02 | -0.07 – 0.11 | -0.06 – 0.10 | 0.612 |  |  |  |  |  |  |  |
| Machiavellianism |  |  |  |  |  |  |  |  |  |  |  | 0.23 | 0.17 | 0.17 – 0.29 | 0.13 – 0.22 | **<0.001** | **<0.001** |  |
| OoT ×  Machiavellianism |  |  |  |  |  |  |  |  |  |  |  | 0.02 | 0.01 | -0.07 – 0.10 | -0.05 – 0.08 | 0.703 | 0.703 |  |
| SC ×  Machiavellianism |  |  |  |  |  |  |  |  |  |  |  | -0.09 | -0.07 | -0.16 – -0.02 | -0.12 – -0.01 | **0.016** | **0.016** |  |
| OoT ×  SC ×  Machiavellianism |  |  |  |  |  |  |  |  |  |  |  | -0.02 | -0.02 | -0.12 – 0.08 | -0.09 – 0.06 | 0.650 | 0.650 |  |
| **Random Effects** | | | | | | | | | | | | | | | | | | |
| σ^2^ | 2.34 | | | | | | 2.34 | | | | | 2.33 | | | | | | |
| τ_00_ | 0.45 _ID:Country_ | | | | | | 0.45 _ID:Country_ | | | | | 0.44 _ID:Country_ | | | | | | |
|  | 0.03 _pic_id_ | | | | | | 0.03 _pic_id_ | | | | | 0.03 _pic_id_ | | | | | | |
|  | 0.01 _Country_ | | | | | | 0.00 _Country_ | | | | | 0.00 _Country_ | | | | | | |
| ICC | 0.17 | | | | | | 0.17 | | | | | 0.17 | | | | | | |
| N | 1888 _ID_ | | | | | | 1889 _ID_ | | | | | 1889 _ID_ | | | | | | |
|  | 4 _Country_ | | | | | | 4 _Country_ | | | | | 4 _Country_ | | | | | | |
|  | 30 _pic_id_ | | | | | | 30 _pic_id_ | | | | | 30 _pic_id_ | | | | | | |
| Observations | 9282 | | | | | | 9287 | | | | | 9287 | | | | | | |
| Marginal R^2^ / Conditional R^2^ | 0.074 / 0.233 | | | | | | 0.073 / 0.231 | | | | | 0.078 / 0.233 | | | | | | |

*Note*. OoT = Occurrence of Tears, SC = Situational Context. Occurrence of tears (-.5 = no tears, .5 = tears); situational context (-.5 = non-manipulative, .5 = manipulative). Continuous predictors are mean-centered.

Further, we explored the impact of the dark personality traits on perceptions of target manipulativeness (Supplementary Table S12). Running the same model with manipulativeness as the outcome variable, we observed a positive association between all three personality traits and perceived manipulativeness. In addition, we observed statistically significant interactions for narcissism and situational context (*β*  = -.06, *p* = .029) and Machiavellianism and situational context (*β*  = -.07, *p* = .016). In general, participants rated targets as more manipulative when these targets were depicted in manipulative than non-manipulative situational contexts, but this difference became smaller with increasing levels of narcissism (Figure S4, N-M) and Machiavellianism (Figure S4, M-M).


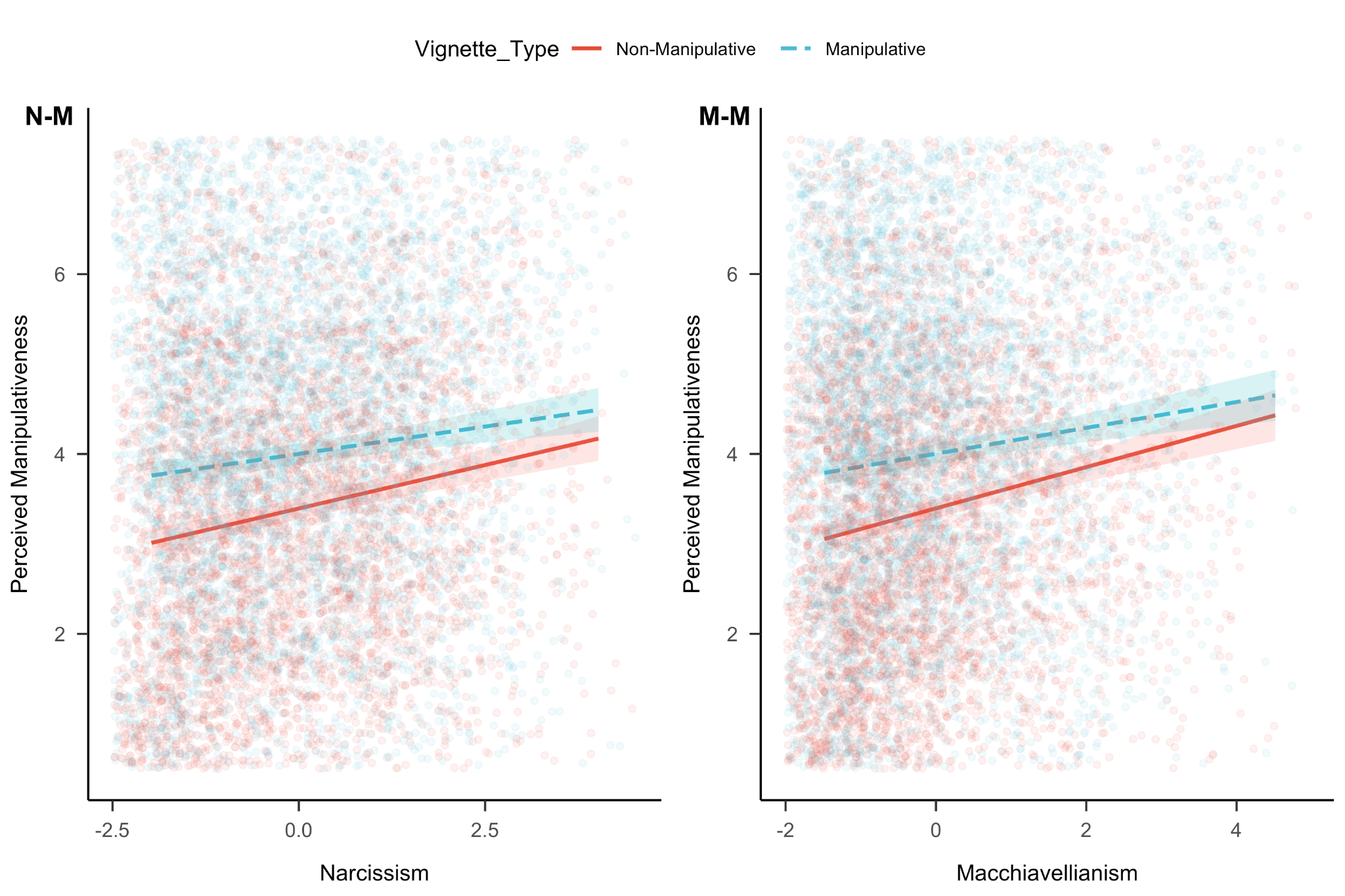


**Supplementary Figure S4.** Interaction plots of narcissism and situational context (N-M) and Machiavellianism and situational context (M-M) for perceived manipulativeness. Confidence bands represent 95% confidence intervals.

**Impact of Target Gender**

We also explored the impact of target gender (i.e., whether the depicted person was a man, -0.5 or a woman, 0.5) on perceptions of honesty, controlling for face warmth and situational contexts and including all two-way interactions. We observed a statistically significant two-way interaction between occurrence of tears and target gender. Tears slightly increased perceptions of honesty for male targets (*d* = .10 [.01, .21]), while they slightly decreased perceptions of honesty for female targets (*d* = -.10 [-.20, .01], Figure S5, H-G). In addition, we observed a statistically significant two-way interaction between situational context and target gender. Manipulative situational contexts reduced perceptions of honesty stronger for male targets (*d* = -.52 [-.62, -.42]) compared to female targets (*d* = -.36 [-.26, -.46]), which was not observed in non-manipulative contexts (Supplementary Table S13).

**Supplementary Table S13.** Multilevel model of occurrence of tears, face warmth, situational context, target gender, and their interactions on perceived honesty.

| *Predictors* | **Perceived Honesty** | | | | |
| --- | --- | --- | --- | --- | --- |
|  | *Estimates* | *std. Beta* | *CI* | *standardized CI* | *p* |
| (Intercept) | 3.95 | 0.02 | 3.71 – 4.19 | -0.14 – 0.17 | **<0.001** |
| Occurrence of Tears | 0.43 | 0.28 | 0.19 – 0.66 | 0.13 – 0.43 | **<0.001** |
| Face Warmth | 0.33 | 0.21 | 0.10 – 0.55 | 0.07 – 0.36 | **0.004** |
| Situational Context | -0.72 | -0.47 | -0.93 – -0.51 | -0.61 – -0.33 | **<0.001** |
| Target Gender | 0.15 | 0.10 | -0.09 – 0.39 | -0.06 – 0.25 | 0.212 |
| OoT x Face Warmth | -0.39 | -0.26 | -0.71 – -0.08 | -0.46 – -0.05 | **0.015** |
| OoT x SC | -0.10 | -0.07 | -0.37 – 0.16 | -0.24 – 0.10 | 0.444 |
| OoT x TG | -0.31 | -0.20 | -0.58 – -0.05 | -0.38 – -0.03 | **0.020** |
| Face Warmth x TG | -0.06 | -0.04 | -0.33 – 0.21 | -0.21 – 0.14 | 0.666 |
| SC x TG | 0.21 | 0.14 | 0.02 – 0.40 | 0.02 – 0.26 | **0.027** |
| Face Warmth x SC | 0.29 | 0.19 | 0.02 – 0.55 | 0.02 – 0.36 | **0.033** |
| OoT x Face Warmth x SC | -0.18 | -0.12 | -0.55 – 0.19 | -0.36 – 0.13 | 0.347 |
| OoT x Face Warmth x TG | 0.11 | 0.07 | -0.27 – 0.48 | -0.17 – 0.31 | 0.576 |
| **Random Effects** | | | | | |
| σ^2^ | 1.69 | | | | |
| τ_00_ _ID:Country_ | 0.52 | | | | |
| τ_00_ _pic_id_ | 0.02 | | | | |
| τ_00_ _Country_ | 0.02 | | | | |
| ICC | 0.25 | | | | |
| N _ID_ | 1883 | | | | |
| N _Country_ | 4 | | | | |
| N _pic_id_ | 30 | | | | |
| Observations | 3720 | | | | |
| Marginal R^2^ / Conditional R^2^ | 0.051 / 0.289 | | | | |

*Note.* OoT = Occurrence of tears, SC = situational context, TG = target gender; Occurrence of tears (-.5 = no tears, .5 = tears); face warmth (-.5 = low warmth, .5 = high warmth); situational context (-.5 = non-manipulative, .5 = manipulative), target gender (-.5 = male, .5 = female).

Similarly, we explored the impact of target gender on ratings of target manipulativeness and expression authenticity, while controlling for face warmth and situational contexts and including all two-way interactions with occurrence of tears (Supplementary Table S14). For target manipulativeness, we observed a significant main effect of target gender with male targets being perceived as more manipulative than female targets (*d* = -.15 [-.25, -.05]). We also observed a statistically significant interaction with occurrence of tears, suggesting that tears increased perceived manipulativeness more strongly for female targets (*d* = .25 [.15, .36]) than for male targets (*d* = .11 [.01, .21]), which was driven by the fact that male targets were perceived as more manipulative when they were presented without tears than with tears (Figure S5, H-M). We observed no statistically significant effect of target gender on perceived expression authenticity.

**Supplementary Table S14.** Multilevel model of occurrence of tears, situational context, face warmth, and target gender and their interactions for perceived target manipulativeness.

| *Predictors* | **Perceived Target Manipulativeness** | | | | |
| --- | --- | --- | --- | --- | --- |
|  | *Estimates* | *std. Beta* | *CI* | *standardized CI* | *p* |
| (Intercept) | 3.71 | -0.10 | 3.51 – 3.91 | -0.22 – 0.01 | **<0.001** |
| Occurrence of Tears | -0.04 | -0.02 | -0.26 – 0.17 | -0.15 – 0.10 | 0.700 |
| Situational Context | 0.63 | 0.36 | 0.48 – 0.78 | 0.28 – 0.45 | **<0.001** |
| Face Warmth | -0.33 | -0.19 | -0.47 – -0.19 | -0.27 – -0.11 | **<0.001** |
| Target Gender | -0.26 | -0.15 | -0.43 – -0.09 | -0.25 – -0.05 | **0.003** |
| OoT × SC | 0.19 | 0.11 | -0.03 – 0.40 | -0.02 – 0.23 | 0.090 |
| OoT x Face Warmth | 0.23 | 0.13 | 0.04 – 0.43 | 0.02 – 0.25 | **0.020** |
| OoT x TG | 0.22 | 0.13 | 0.01 – 0.43 | 0.00 – 0.25 | **0.043** |
| **Random Effects** | | | | | |
| σ^2^ | 2.36 | | | | |
| τ_00_ _ID:Country_ | 0.49 | | | | |
| τ_00_ _pic_id_ | 0.01 | | | | |
| τ_00_ _Country_ | 0.01 | | | | |
| ICC | 0.18 | | | | |
| N _ID_ | 1883 | | | | |
| N _Country_ | 4 | | | | |
| N _pic_id_ | 30 | | | | |
| Observations | 3723 | | | | |
| Marginal R^2^ / Conditional R^2^ | 0.058 / 0.225 | | | | |

*Note.* OoT = Occurrence of tears, SC = situational context, TG = target gender; Occurrence of tears (-.5 = no tears, .5 = tears); face warmth (-.5 = low warmth, .5 = high warmth); situational context (-.5 = non-manipulative, .5 = manipulative), target gender (-.5 = male, .5 = female).

We also tested the impact of target gender for perceived authenticity, but observed no significant main effects or interaction effects of target gender (Supplementary Table S15). Finally, we tested the impact of target gender for the relationship between occurrence of tears and support intentions (Supplementary Table S16). We observed both a statistically significant main effect of tears and an interaction effect between tears and target gender. Tears, in contrast to no tears, increased support intentions for male targets (*d* = .18 [.07, .30]), but not for female targets (*d* = -.05 [-.17, .07], Figure S5, S-G). When testing the mediation by perceived honesty separately for male and female targets, we observed that perceived honesty showed a stronger mediation effect for male (*B* = .32 [.09, .56]) compared to female targets (*B* = .12 [-.09, .34]).

**Supplementary Table S15.** Multilevel model of occurrence of tears, situational context, face warmth, and target gender and their interactions for perceived authenticity.

| *Predictors* | **Perceived Authenticity** | | | | |
| --- | --- | --- | --- | --- | --- |
|  | *Estimates* | *std. Beta* | *CI* | *standardized CI* | *p* |
| (Intercept) | 4.35 | 0.12 | 4.11 – 4.60 | -0.03 – 0.26 | **<0.001** |
| Occurrence of Tears | 0.22 | 0.13 | 0.01 – 0.43 | 0.01 – 0.26 | **0.040** |
| Situational Context | -0.34 | -0.20 | -0.49 – -0.20 | -0.29 – -0.12 | **<0.001** |
| Face Warmth | 0.02 | 0.01 | -0.11 – 0.16 | -0.07 – 0.09 | 0.739 |
| Target Gender | 0.04 | 0.02 | -0.18 – 0.25 | -0.10 – 0.15 | 0.727 |
| OoT ×  SC | -0.29 | -0.17 | -0.50 – -0.08 | -0.30 – -0.05 | **0.006** |
| OoT x Face Warmth | -0.25 | -0.15 | -0.44 – -0.06 | -0.26 – -0.03 | **0.010** |
| OoT x TG | -0.10 | -0.06 | -0.31 – 0.11 | -0.18 – 0.06 | 0.338 |
| **Random Effects** | | | | | |
| σ^2^ | 2.13 | | | | |
| τ_00_ _ID:Country_ | 0.60 | | | | |
| τ_00_ _pic_id_ | 0.05 | | | | |
| τ_00_ _Country_ | 0.03 | | | | |
| ICC | 0.24 | | | | |
| N _ID_ | 1883 | | | | |
| N _Country_ | 4 | | | | |
| N _pic_id_ | 30 | | | | |
| Observations | 3724 | | | | |
| Marginal R^2^ / Conditional R^2^ | 0.026 / 0.260 | | | | |

*Note.* OoT = Occurrence of tears, SC = situational context, TG = target gender; Occurrence of tears (-.5 = no tears, .5 = tears); face warmth (-.5 = low warmth, .5 = high warmth); situational context (-.5 = non-manipulative, .5 = manipulative), target gender (-.5 = male, .5 = female).

**Supplementary Table S16.** Multilevel model with occurrence of tears and target gender and their interaction for support intentions.

|  | **Support Intentions** | | | | |
| --- | --- | --- | --- | --- | --- |
| *Predictors* | *Estimates* | *std. Beta* | *CI* | *standardized CI* | *p* |
| (Intercept) | 3.62 | -0.14 | 3.37 – 3.88 | -0.29 – 0.01 | **<0.001** |
| Occurrence of Tears | 0.25 | 0.15 | 0.09 – 0.41 | 0.05 – 0.24 | **0.002** |
| Target Gender | 0.39 | 0.23 | 0.19 – 0.59 | 0.11 – 0.34 | **<0.001** |
| Occurrence of Tears × Target  Gender | -0.32 | -0.19 | -0.53 – -0.12 | -0.31 – -0.07 | **0.002** |
| **Random Effects** | | | | | |
| σ^2^ | 1.93 | | | | |
| τ_00_ _ID:Country_ | 0.90 | | | | |
| τ_00_ _pic_id_ | 0.04 | | | | |
| τ_00_ _Country_ | 0.05 | | | | |
| ICC | 0.34 | | | | |
| N _ID_ | 1883 | | | | |
| N _Country_ | 4 | | | | |
| N _pic_id_ | 30 | | | | |
| Observations | 3724 | | | | |
| Marginal R^2^ / Conditional R^2^ | 0.007 / 0.342 | | | | |

*Note.* Occurrence of Tears (-0.5 = no tears, 0.5 = tears), target gender (-0.5 = male, 0.5 = female).

**Impact of Perceived Appropriateness.** Next, we performed exploratory analyses on the impact of perceived expression appropriateness. First, we ran a multilevel model with perceived appropriateness as the outcome variable and occurrence of tears, face warmth, and situational context as predictors, including their interactions (Supplementary Table S17). We found a statistically significant main effect of situational context indicating that expressions of the targets depicted in non-manipulative contexts were perceived as more appropriate than expressions of the targets depicted in manipulative ones (*d* = -.41 [-.54, -.28]). We also observed a statistically significant three-way interaction. Expressions of the targets who were high in warmth were perceived as more appropriate when they were not shedding tears, while this effect was reversed for targets low in warmth. This interaction was stronger for manipulative contexts compared to non-manipulative contexts in which appropriateness ratings were highly similar across all combinations (Figure S5).

**Supplementary Table S17.** Multilevel model with occurrence of tears, face warmth, situational context, and their interactions for perceived expression appropriateness.

|  | **Perceived Expression Appropriateness** | | | | |
| --- | --- | --- | --- | --- | --- |
| *Predictors* | *Estimates* | *std. Beta* | *CI* | *standardized CI* | *p* |
| (Intercept) | 4.33 | 0.15 | 4.07 – 4.59 | 0.01 – 0.30 | **<0.001** |
| Occurrence of Tears | 0.09 | 0.05 | -0.13 – 0.32 | -0.08 – 0.18 | 0.419 |
| Face Warmth | 0.01 | 0.00 | -0.22 – 0.24 | -0.12 – 0.13 | 0.950 |
| Situational Context | -0.74 | -0.41 | -0.96 – -0.51 | -0.54 – -0.28 | **<0.001** |
| OoT x Face Warmth | -0.24 | -0.13 | -0.56 – 0.09 | -0.31 – 0.05 | 0.156 |
| OoT x SC | 0.17 | 0.09 | -0.15 – 0.49 | -0.08 – 0.27 | 0.304 |
| Face Warmth x SC | 0.50 | 0.28 | 0.18 – 0.81 | 0.10 – 0.45 | **0.002** |
| OoT x Face Warmth x SC | -0.49 | -0.27 | -0.94 – -0.04 | -0.52 – -0.02 | **0.035** |
| **Random Effects** | | | | | |
| σ^2^ | 3.06 | | | | |
| τ_00_ _pic_id_ | 0.05 | | | | |
| τ_00_ _Country_ | 0.04 | | | | |
| ICC | 0.03 | | | | |
| N _Country_ | 4 | | | | |
| N _pic_id_ | 30 | | | | |
| Observations | 3724 | | | | |
| Marginal R^2^ / Conditional R^2^ | 0.029 / 0.055 | | | | |

*Note.* OoT = occurrence of tears, SC = situational context. Occurrence of tears (-.5 = no tears, .5 = tears), face warmth (-.5 = low warmth, .5 = high warmth), situational context (-.5 = non-manipulative, .5 = manipulative).


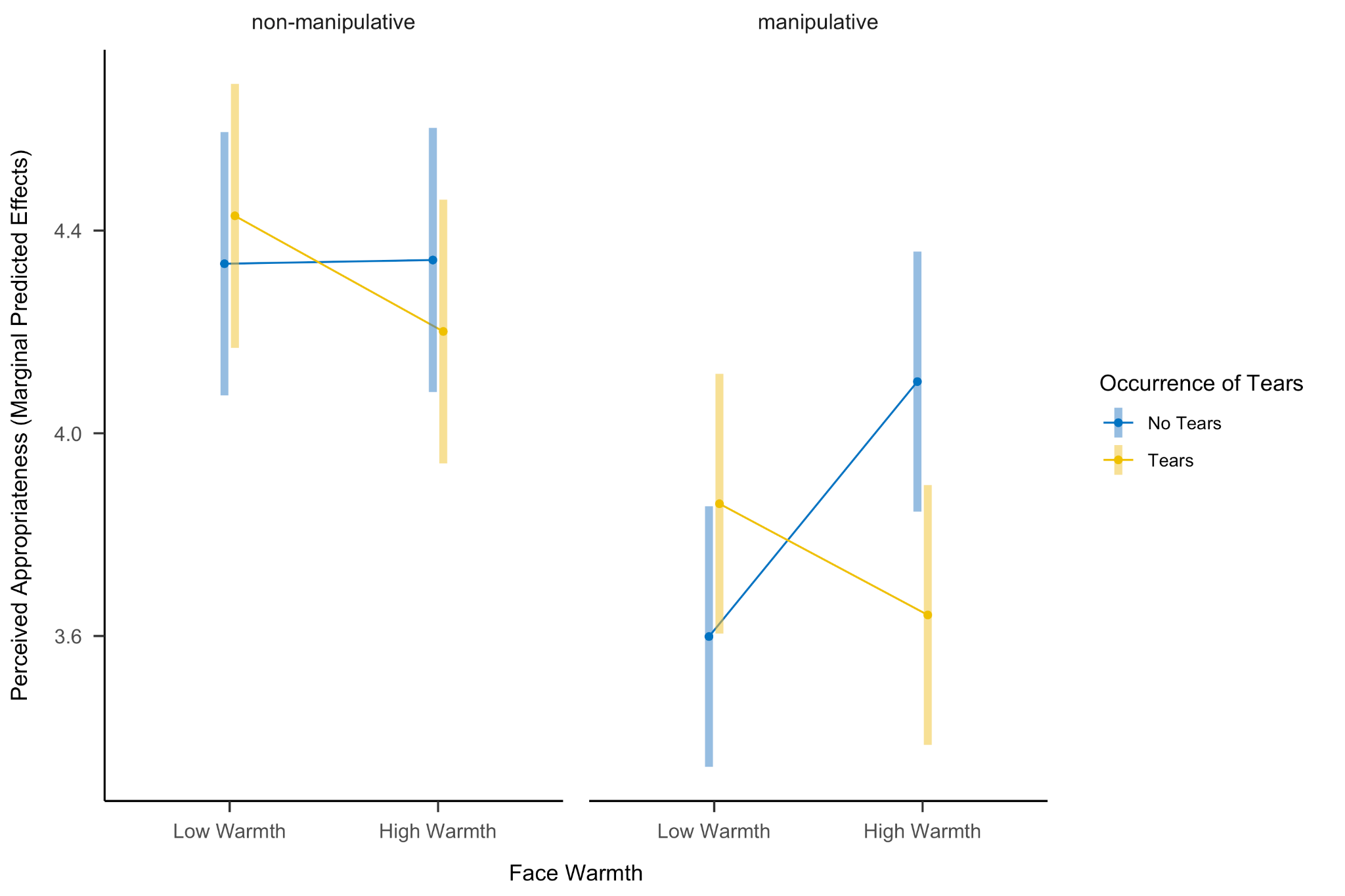


**Supplementary Figure S5.** Overview of Three-Way Interaction among Occurrence of Tears, Face Warmth, and Situational Context on Perceived Appropriateness. Error Bars Represent 95% Confidence Intervals.

In addition, we explored the moderation effect of perceived expression appropriateness by running four models with perceived honesty, manipulativeness, expression authenticity, and support intentions as the DVs and occurrence of tears, perceived appropriateness (centered), and situational context as predictors, including all possible interactions (Supplementary Table S18). For all measures, we observed a statistically significant main effect of perceived appropriateness. Higher appropriateness was associated with higher perceived honesty, authenticity and support intentions and lower perceived manipulativeness. Further, for perceived honesty we observed a statistically significant three-way interaction among tears, appropriateness, and situational context. Tears increased ratings of honesty for higher levels of perceived appropriateness but only for manipulative contexts (Figure S6, A). For perceived manipulativeness, we observed that all effects were statistically significant. The significant three-way interaction suggested that targets shedding tears (compared to targets with no tears) were perceived as less manipulative for higher levels of perceived appropriateness, but this effect was strongest for manipulative contexts. Put differently, targets without tears were perceived as the more manipulative, the less appropriate their expression was perceived, but this effect was significantly stronger when targets shed tears (Figure S6, B). For perceived expression authenticity, we also observed a statistically significant three-way interaction with tears being perceived as less authentic for low levels of appropriateness, and more strongly so in manipulative contexts (Figure S6, C). Finally, for support intentions, we did not observe a statistically significant three-way interaction, but a significant interaction between occurrence of tears and perceived expression appropriateness. Tears resulted in slightly stronger support intentions for high levels of appropriateness compared to no tears (Figure S6, D).

**Supplementary Table S18.** Multilevel models with occurrence of tears, situational context, and perceived appropriateness (centered) for perceived honesty, perceived target manipulativeness, perceived authenticity, and support intentions.

|  | **Perceived Honesty** | | | **Perceived Manipulativeness** | | | **Perceived Authenticity** | | | **Support Intentions** | | |
| --- | --- | --- | --- | --- | --- | --- | --- | --- | --- | --- | --- | --- |
| *Predictors* | *Estimates* | *CI* | *p* | *Estimates* | *CI* | *p* | *Estimates* | *CI* | *p* | *Estimates* | *CI* | *p* |
| (Intercept) | 4.08 | 4.00 – 4.16 | **<0.001** | 3.45 | 3.33 – 3.57 | **<0.001** | 4.23 | 4.17 – 4.29 | **<0.001** | 3.98 | 3.81 – 4.16 | **<0.001** |
| Occurrence of Tears | 0.07 | 0.00 – 0.14 | **0.039** | 0.17 | 0.08 – 0.27 | **<0.001** | 0.02 | -0.04 – 0.09 | 0.509 | 0.15 | 0.07 – 0.23 | **<0.001** |
| Perceived Appropriateness | 0.56 | 0.54 – 0.59 | **<0.001** | -0.18 | -0.22 – -0.14 | **<0.001** | 0.72 | 0.70 – 0.75 | **<0.001** | 0.48 | 0.45 – 0.51 | **<0.001** |
| Situational Context | -0.26 | -0.32 – -0.19 | **<0.001** | 0.54 | 0.44 – 0.63 | **<0.001** | -0.08 | -0.14 – -0.01 | **0.023** | -0.28 | -0.36 – -0.20 | **<0.001** |
| OoT x Appropriateness | 0.01 | -0.03 – 0.05 | 0.644 | -0.09 | -0.14 – -0.04 | **0.001** | -0.03 | -0.07 – 0.00 | 0.084 | 0.08 | 0.04 – 0.12 | **<0.001** |
| OoT x SC | -0.07 | -0.16 – 0.02 | 0.139 | 0.18 | 0.05 – 0.32 | **0.008** | -0.12 | -0.21 – -0.03 | **0.011** | -0.08 | -0.19 – 0.03 | 0.153 |
| SC x Appropriateness | -0.03 | -0.07 – 0.01 | 0.142 | 0.14 | 0.08 – 0.19 | **<0.001** | -0.06 | -0.09 – -0.02 | **0.003** | 0.00 | -0.04 – 0.05 | 0.972 |
| OoT x SC x Appropriateness | 0.09 | 0.04 – 0.14 | **0.001** | -0.20 | -0.27 – -0.12 | **<0.001** | 0.10 | 0.05 – 0.16 | **<0.001** | 0.04 | -0.02 – 0.10 | 0.186 |
| **Random Effects** | | | | | | | | | | | | |
| σ^2^ | 1.25 | | | 2.67 | | | 1.25 | | | 1.83 | | |
| τ_00_ | 0.00 _pic_id_ | | | 0.01 _pic_id_ | | | 0.01 _pic_id_ | | | 0.02 _pic_id_ | | |
|  | 0.00 _Country_ | | | 0.01 _Country_ | | | 0.00 _Country_ | | | 0.03 _Country_ | | |
| ICC | 0.01 | | | 0.01 | | |  | | | 0.02 | | |
| N | 4 _Country_ | | | 4 _Country_ | | | 4 _Country_ | | | 4 _Country_ | | |
|  | 30 _pic_id_ | | | 30 _pic_id_ | | | 30 _pic_id_ | | | 30 _pic_id_ | | |
| Observations | 9294 | | | 9296 | | | 9298 | | | 9298 | | |
| Marginal R^2^ / Conditional R^2^ | 0.471 / 0.474 | | | 0.112 / 0.119 | | | 0.561 / NA | | | 0.344 / 0.360 | | |

*Note.* OoT = Occurrence of tears, SC = situational context. Occurrence of tears (-.5 = no tears, .5 = tears), situational context (-.5 = non-manipulative, .5 = manipulative). Appropriateness is mean centered.


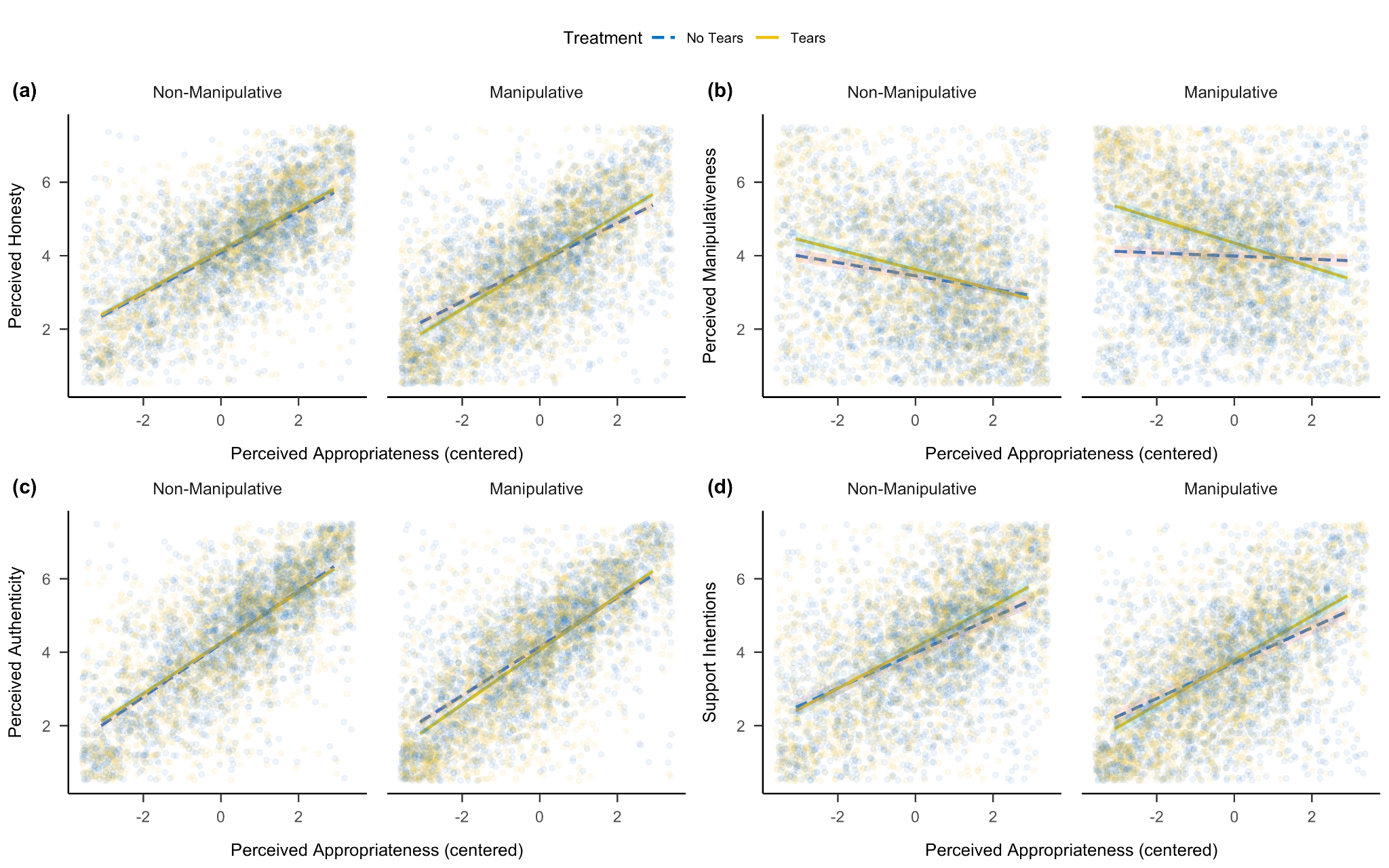


**Supplementary Figure S6.** Overview of Three-Way Interactions of Occurrence of Tears, Situational Context and Perceived Appropriateness (Centered) on (a) Perceived honesty, (b) Perceived Manipulativeness, (c) Perceived Authenticity, and (d) Support Intentions. Confidence bands represent 95% confidence intervals

**Country Effects**

**Supplementary Table S19.** Multilevel model with occurrence of tears, face warmth, situational context, country trust and their interactions for perceived honesty.

|  | **Perceived Honesty** | | | | |
| --- | --- | --- | --- | --- | --- |
| *Predictors* | *Estimates* | *std. Beta* | *CI* | *standardized CI* | *p* |
| (Intercept) | 4.02 | 0.06 | 3.84 – 4.20 | -0.05 – 0.18 | **<0.001** |
| Occurrence of Tears | 0.26 | 0.17 | 0.02 – 0.49 | 0.01 – 0.32 | **0.032** |
| Face Warmth | 0.27 | 0.18 | 0.08 – 0.47 | 0.05 – 0.30 | **0.007** |
| Situational Context | -0.72 | -0.47 | -0.95 – -0.50 | -0.62 – -0.32 | **<0.001** |
| Country Trust | -0.02 | -0.01 | -0.24 – 0.20 | -0.16 – 0.13 | 0.866 |
| OoT x Face Warmth | -0.33 | -0.21 | -0.59 – -0.07 | -0.38 – -0.05 | **0.012** |
| OoT x SC | -0.01 | -0.01 | -0.33 – 0.31 | -0.21 – 0.20 | 0.961 |
| OoT × Country  Trust | 0.03 | 0.02 | -0.26 – 0.32 | -0.17 – 0.21 | 0.819 |
| Face Warmth x Country Trust | 0.05 | 0.03 | -0.12 – 0.22 | -0.08 – 0.14 | 0.555 |
| SC x Country Trust | 0.23 | 0.15 | -0.04 – 0.49 | -0.03 – 0.32 | 0.095 |
| Face Warmth x SC | 0.29 | 0.19 | 0.02 – 0.55 | 0.01 – 0.36 | **0.033** |
| OoT x Face Warmth x SC | -0.19 | -0.12 | -0.56 – 0.19 | -0.37 – 0.12 | 0.323 |
| OoT x SC x Country Trust | -0.19 | -0.13 | -0.57 – 0.18 | -0.37 – 0.12 | 0.314 |
| **Random Effects** | | | | | |
| σ^2^ | 1.70 | | | | |
| τ_00_ _ID_ | 0.53 | | | | |
| τ_00_ _pic_id_ | 0.02 | | | | |
| ICC | 0.25 | | | | |
| N _ID_ | 1883 | | | | |
| N _pic_id_ | 30 | | | | |
| Observations | 3720 | | | | |
| Marginal R^2^ / Conditional R^2^ | 0.048 / 0.283 | | | | |

*Note.* OoT = occurrence of tears, SC = situational context. Occurrence of tears (-.5 = no tears, .5 = tears), face warmth (-.5 = low warmth, .5 = high warmth), situational context (-.5 = non-manipulative, .5 = manipulative), country trust (-.5 = low trust, .5 = high trust).

**Supplementary Table S20.** Multilevel model with occurrence of tears, country trust, and their interaction for perceived honesty, perceived target manipulativeness, perceived authenticity, and support intentions.

|  | **Perceived Honesty** | | | **Perceived Target Manipulativeness** | | | **Perceived Authenticity** | | | **Support Intentions** | | | |
| --- | --- | --- | --- | --- | --- | --- | --- | --- | --- | --- | --- | --- | --- |
| *Predictors* | *Estimates* | *CI* | *p* | *Estimates* | *CI* | *p* | *Estimates* | *CI* | *p* | *Estimates* | *CI* | *p* |  |
| (Intercept) | 3.94 | 3.83 – 4.05 | **<0.001** | 3.71 | 3.61 – 3.82 | **<0.001** | 4.21 | 4.09 – 4.33 | **<0.001** | 3.89 | 3.75 – 4.02 | **<0.001** |  |
| Occurrence of Tears | -0.05 | -0.16 – 0.07 | 0.443 | 0.45 | 0.33 – 0.57 | **<0.001** | -0.17 | -0.29 – -0.04 | **0.010** | 0.08 | -0.07 – 0.22 | 0.308 |  |
| Country Trust | 0.11 | -0.01 – 0.23 | 0.084 | -0.02 | -0.15 – 0.11 | 0.750 | 0.09 | -0.04 – 0.22 | 0.173 | -0.01 | -0.16 – 0.14 | 0.889 |  |
| Occurrence of Tears x Country Trust | -0.04 | -0.21 – 0.13 | 0.661 | -0.26 | -0.44 – -0.08 | **0.005** | 0.03 | -0.16 – 0.21 | 0.776 | -0.09 | -0.30 – 0.12 | 0.378 |  |
| **Random Effects** | | | | | | | | | | | | | |
| σ^2^ | 1.87 | | | 2.49 | | | 2.25 | | | 1.87 | | | |
| τ_00_ | 0.50 _ID_ | | | 0.48 _ID_ | | | 0.58 _ID_ | | | 0.97 _ID_ | | | |
|  | 0.04 _pic_id_ | | | 0.03 _pic_id_ | | | 0.05 _pic_id_ | | | 0.06 _pic_id_ | | | |
| ICC | 0.22 | | | 0.17 | | | 0.22 | | | 0.36 | | | |
| N | 1893 _ID_ | | | 1893 _ID_ | | | 1893 _ID_ | | | 1893 _ID_ | | | |
|  | 30 _pic_id_ | | | 30 _pic_id_ | | | 30 _pic_id_ | | | 30 _pic_id_ | | | |
| Observations | 9294 | | | 9296 | | | 9298 | | | 9298 | | | |
| Marginal R^2^ / Conditional R^2^ | 0.001 / 0.223 | | | 0.012 / 0.180 | | | 0.003 / 0.220 | | | 0.001 / 0.356 | | | |

*Note.* Occurrence of tears (-.5 = no tears, .5 = tears), country trust (-.5 = low trust, .5 = high trust).

**Presentation Order**

**Supplementary Table S21.** Multilevel model of occurrence of tears, face warmth, situational context, and presentation order and their interactions.

|  | **Perceived Honesty** | | | **Perceived Authenticity** | | | **Support Intentions** | | |
| --- | --- | --- | --- | --- | --- | --- | --- | --- | --- |
| *Predictors* | *Estimates* | *CI* | *p* | *Estimates* | *CI* | *p* | *Estimates* | *CI* | *p* |
| (Intercept) | 4.00 | 3.71 – 4.28 | **<0.001** | 4.33 | 4.01 – 4.65 | **<0.001** | 4.27 | 3.93 – 4.60 | **<0.001** |
| Occurrence of Tears | 0.48 | 0.20 – 0.75 | **0.001** | 0.45 | 0.15 – 0.75 | **0.004** | 0.59 | 0.30 – 0.88 | **<0.001** |
| Face Warmth | 0.27 | 0.00 – 0.54 | **0.047** | -0.24 | -0.54 – 0.07 | 0.125 | 0.24 | -0.05 – 0.52 | 0.106 |
| Situational Order | -0.49 | -0.76 – -0.21 | **<0.001** | -0.33 | -0.64 – -0.03 | **0.032** | -0.50 | -0.78 – -0.21 | **0.001** |
| Presentation Order | 0.01 | -0.06 – 0.08 | 0.770 | 0.05 | -0.03 – 0.12 | 0.200 | -0.13 | -0.20 – -0.06 | **<0.001** |
| OoT x Presentation Order | -0.07 | -0.14 – -0.01 | **0.032** | -0.12 | -0.20 – -0.05 | **0.001** | -0.07 | -0.14 – -0.00 | **0.041** |
| Face Warmth x Presentation Order | 0.01 | -0.06 – 0.08 | 0.825 | 0.02 | -0.05 – 0.10 | 0.587 | 0.09 | 0.01 – 0.16 | **0.019** |
| SC x Presentation Order | -0.04 | -0.11 – 0.02 | 0.201 | -0.07 | -0.14 – 0.00 | 0.066 | -0.04 | -0.11 – 0.03 | 0.245 |
| OoT x Face Warmth | -0.33 | -0.58 – -0.07 | **0.013** | -0.06 | -0.35 – 0.22 | 0.663 | -0.50 | -0.77 – -0.24 | **<0.001** |
| OoT x SC | -0.09 | -0.35 – 0.17 | 0.507 | -0.10 | -0.40 – 0.19 | 0.495 | -0.08 | -0.36 – 0.20 | 0.561 |
| Face Warmth x SC | 0.29 | 0.02 – 0.55 | **0.034** | 0.38 | 0.09 – 0.68 | **0.010** | 0.10 | -0.18 – 0.38 | 0.470 |
| OoT x Face Warmth x SC | -0.19 | -0.56 – 0.18 | 0.317 | -0.35 | -0.77 – 0.06 | 0.094 | 0.04 | -0.35 – 0.44 | 0.829 |
| **Random Effects** | | | | | | | | | |
| σ^2^ | 1.70 | | | 2.11 | | | 1.71 | | |
| τ_00_ | 0.52 _ID:Country_ | | | 0.60 _ID:Country_ | | | 0.95 _ID:Country_ | | |
|  | 0.02 _pic_id_ | | | 0.05 _pic_id_ | | | 0.04 _pic_id_ | | |
|  | 0.02 _Country_ | | | 0.03 _Country_ | | | 0.05 _Country_ | | |
| ICC | 0.25 | | | 0.24 | | | 0.38 | | |
| N | 1883 _ID_ | | | 1883 _ID_ | | | 1883 _ID_ | | |
|  | 4 _Country_ | | | 4 _Country_ | | | 4 _Country_ | | |
|  | 30 _pic_id_ | | | 30 _pic_id_ | | | 30 _pic_id_ | | |
| Observations | 3720 | | | 3724 | | | 3724 | | |
| Marginal R^2^ / Conditional R^2^ | 0.049 / 0.288 | | | 0.032 / 0.266 | | | 0.063 / 0.416 | | |

*Note.* OoT = occurrence of tears, SC = situational context. Occurrence of tears (-.5 = no tears, .5 = tears), face warmth (-.5 = low warmth, .5 = high warmth), situational context (-.5 = non-manipulative, .5 = manipulative).

**
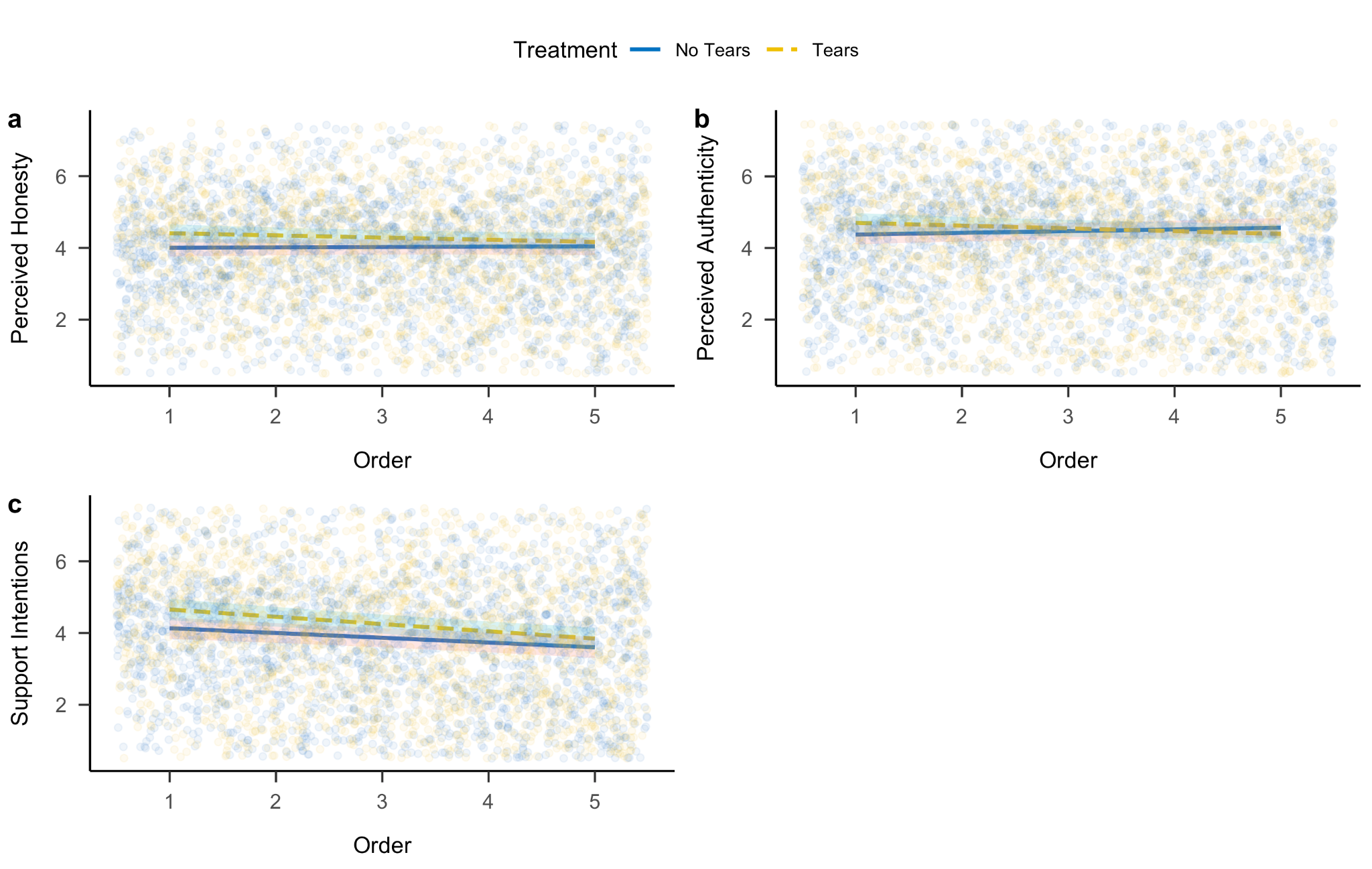
**

**Supplementary Figure S7.** Interaction effects of occurrence of tears and order for perceived honesty (a), perceived authenticity (b), and support intentions (c). Confidence bands represent 95% confidence intervals.
