# Supplementary material for: The honesty behind tears: Situational, individual, and cultural influences on the perception of emotional tears as sincere
Source: PLoS One. 2025 Jul 16;20(7):e0324954. doi: 10.1371/journal.pone.0324954 (PMC12266444; doi:10.1371/journal.pone.0324954)
Supplement: S5 Note — (DOCX) [file pone.0324954.s005.docx]

**Supplementary Note S5**

**Study 2 Additional Analyses**

**Descriptive Statistics**

**Supplementary Table S22.** Descriptive statistics for measures in Study 2 across all treatments and for occurrence of tears separately.

| **Variable** | **Overall** | | | | **No Tears** | | | **Tears** | | |
| --- | --- | --- | --- | --- | --- | --- | --- | --- | --- | --- |
|  | *r/a* | *N* | *M* | *SD* | *n* | *M* | *SD* | *n* | *M* | *SD* |
| Perceived Appropriateness | - | 1595 | 4.62 | 1.6 | 779 | 4.57 | 1.58 | 816 | 4.66 | 1.61 |
| Perceived Authenticity | 0.88 | 1594 | 4.63 | 1.6 | 778 | 4.58 | 1.55 | 816 | 4.68 | 1.65 |
| Perceived Target Manipulativeness | - | 1595 | 3.4 | 1.58 | 779 | 3.36 | 1.56 | 816 | 3.43 | 1.61 |
| Perceived Competence | 0.80 | 1594 | 4.37 | 1.2 | 779 | 4.47 | 1.18 | 815 | 4.27 | 1.2 |
| Perceived Warmth | 0.81 | 1595 | 3.99 | 1.38 | 779 | 3.91 | 1.38 | 816 | 4.07 | 1.38 |
| Perceived Honesty | 0.83 | 1595 | 4.49 | 1.41 | 779 | 4.41 | 1.36 | 816 | 4.57 | 1.46 |
| Perceived Helplessness | - | 1595 | 3.68 | 1.61 | 779 | 3.18 | 1.55 | 816 | 4.15 | 1.53 |
| Empathic Concern | 0.87 | 1595 | 4.07 | 1.63 | 779 | 3.73 | 1.56 | 816 | 4.39 | 1.63 |
| Personal Distress | 0.53 | 1595 | 2.82 | 1.45 | 779 | 2.36 | 1.31 | 816 | 3.27 | 1.43 |
| Support Intentions | - | 1595 | 4.59 | 1.56 | 779 | 4.45 | 1.54 | 816 | 4.72 | 1.58 |
| Psychopathy | 0.80 | 1592 | 2.41 | 1.16 | 778 | 2.37 | 1.14 | 814 | 2.46 | 1.18 |
| Narcissism | 0.86 | 1591 | 2.53 | 1.24 | 777 | 2.53 | 1.24 | 814 | 2.54 | 1.23 |
| Machiavellianism | 0.86 | 1592 | 2.15 | 1.1 | 778 | 2.14 | 1.1 | 814 | 2.16 | 1.09 |

**Supplementary Table S23.** Linear regression model for occurrence of tears, target gender, situational context and their interactions for perceived honesty in Study 2.

| *Predictors* | **Perceived Honesty** | | | | |
| --- | --- | --- | --- | --- | --- |
|  | *Estimates* | *std. Beta* | *CI* | *standardized CI* | *p* |
| (Intercept) | 4.64 | 0.10 | 4.42 – 4.85 | -0.05 – 0.25 | **<0.001** |
| Occurrence of Tears | 0.12 | 0.09 | -0.16 – 0.41 | -0.12 – 0.29 | 0.397 |
| Target Gender | 0.14 | 0.10 | -0.14 – 0.43 | -0.10 – 0.30 | 0.332 |
| Situational Context | -0.70 | -0.50 | -0.99 – -0.41 | -0.70 – -0.29 | **<0.001** |
| OoT × TG | -0.07 | -0.05 | -0.46 – 0.33 | -0.33 – 0.23 | 0.740 |
| OoT x SC | 0.12 | 0.09 | -0.27 – 0.51 | -0.19 – 0.36 | 0.550 |
| TG x SC | 0.27 | 0.19 | -0.12 – 0.66 | -0.09 – 0.47 | 0.178 |
| OoT x TG x SC | 0.00 | 0.00 | -0.55 – 0.55 | -0.39 – 0.39 | 0.999 |
| Observations | 1595 | | | | |
| R^2^ / R^2^ adjusted | 0.045 / 0.041 | | | | |

*Note.* OoT = Occurrence of tears, TG = target gender, SC = social context. Occurrence of tears (-.5 no tears, .5 tears), target gender (-.5 = male, .5 = female), social context (-.5 = non-manipulative, .5 = manipulative).

**Supplementary Table S24.** Linear regression with occurrence of tears, target gender, and situational context for perceived honesty in Study 2.

| *Predictors* | **Perceived Honesty** | | | | |
| --- | --- | --- | --- | --- | --- |
|  | *Estimates* | *std. Beta* | *CI* | *standardized CI* | *p* |
| (Intercept) | 4.54 | 0.04 | 4.40 – 4.69 | -0.06 – 0.14 | **<0.001** |
| Occurrence of Tears | 0.16 | 0.11 | 0.02 – 0.29 | 0.01 – 0.21 | **0.024** |
| Target Gender | 0.25 | 0.18 | 0.11 – 0.39 | 0.08 – 0.27 | **<0.001** |
| Situational Context | -0.50 | -0.35 | -0.64 – -0.36 | -0.45 – -0.26 | **<0.001** |
| Observations | 1595 | | | | |
| R^2^ / R^2^ adjusted | 0.043 / 0.041 | | | | |

*Note.* Occurrence of tears (-.5 no tears, .5 tears), target gender (-.5 = male, .5 = female), social context (-.5 = non-manipulative, .5 = manipulative).

**Mediation Via Manipulativeness and Authenticity (H2)**

**Supplementary Table S25.** Overview of mediation coefficients for H2 and H3 in Study.

| Outcome | Moderator | Moderator Levels | a1 | a2 | b1 | b2 | c’ |
| --- | --- | --- | --- | --- | --- | --- | --- |
|  |  |  | *B* (*SE*) | | | | |
| Perceived Honesty |  |  | OoT 🡪 Manipulativeness | OoT 🡪 Authenticity | Manipulativeness 🡪 Honesty) | Authenticity 🡪 Honesty | OoT 🡪 Honesty |
|  | Target Gender | Female | 0.14 (0.11) | 0.07 (0.12) | -0.27** (0.03) | 0.51** (0.03) | 0.11 (0.06) |
|  |  | Male | -0.02 (0.11) | 0.15 (0.11) | -0.27** (0.03) | 0.53** (0.03) | 0.14 (0.07) |
|  | Situational Context | Non-Manipulative | 0.03 (0.11) | 0.05 (0.11) | -0.24** (0.03) | 0.51** (0.03) | 0.07 (0.06) |
|  |  | Manipulative | 0.13 (0.11) | 0.14 (0.11) | -0.30** (0.03) | 0.53** (0.03) | 0.17* (0.06) |
|  | None |  | 0.07 (0.08) | 0.10 (0.08) | -0.28 (0.02)** | 0.52 (0.02)** | 0.12 (0.05)* |
| Support Intentions |  |  | OoT 🡪 Honesty | - | Honesty 🡪 Support Intentions | - | OoT 🡪 Support Intentions |
|  | Target Gender | Female | 0.11 (0.10) | - | 0.75 (0.03)** | - | 0.09 (0.08) |
|  |  | Male | 0.22 (0.10) | - | 0.78 (0.03)** | - | 0.21 (0.08)* |
|  | Situational Context | Non-Manipulative | 0.08 (0.09) | - | 0.68 (0.03)** | - | 0.21 (0.08)* |
|  |  | Manipulative | 0.21 (0.10) | - | 0.79 (0.03)** | - | 0.08 (0.08) |
|  | None |  | 0.16 (0.07) | - | 0.77 (0.02)** | - | 0.15 (0.06)* |

*Note.* * < 0.025, ** < 0.001. OoT = Occurrence of Tears. All coefficients are unstandardized.

**Supplementary Table S26.** Interaction between occurrence of tears, target gender and situational context for support intentions. Positive effects indicate higher support intentions for tearful pictures.

| Target Gender | Situational Context | *d* | 95% CI |
| --- | --- | --- | --- |
| Female | Non-Manipulative | 0.23 | 0.03 – 0.43 |
| Male | Non-Manipulative | 0.16 | -0.05 – 0.36 |
| Female | Manipulative | 0.04 | -0.14 – 0.23 |
| Male | Manipulative | 0.28 | 0.07 – 0.47 |

**Supplementary Table S27.** Linear regression of occurrence of tears, target gender, and situational context for support intentions in Study 2.

|  | **Support Intentions** | | | | |
| --- | --- | --- | --- | --- | --- |
| *Predictors* | *Estimates* | *std. Beta* | *CI* | *standardized CI* | *p* |
| (Intercept) | 4.80 | 0.13 | 4.56 – 5.03 | -0.02 – 0.28 | **<0.001** |
| Occurrence of Tears | 0.22 | 0.14 | -0.09 – 0.54 | -0.06 – 0.34 | 0.162 |
| Target Gender | 0.06 | 0.04 | -0.25 – 0.37 | -0.16 – 0.24 | 0.692 |
| Situational Context | -0.97 | -0.62 | -1.28 – -0.65 | -0.82 – -0.42 | **<0.001** |
| OoT × TG | 0.10 | 0.06 | -0.33 – 0.53 | -0.21 – 0.34 | 0.656 |
| OoT x SC | 0.21 | 0.14 | -0.22 – 0.64 | -0.14 – 0.41 | 0.336 |
| TG x SC | 0.48 | 0.31 | 0.06 – 0.91 | 0.04 – 0.58 | **0.027** |
| OoT x TG x SC | -0.47 | -0.30 | -1.06 – 0.13 | -0.68 – 0.08 | 0.122 |
| Observations | 1595 | | | | |
| R^2^ / R^2^ adjusted | 0.072 / 0.068 | | | | |

*Note.* OoT = Occurrence of tears, TG = target gender, SC = social context. Occurrence of tears (-.5 no tears, .5 tears), target gender (-.5 = male, .5 = female), social context (-.5 = non-manipulative, .5 = manipulative).

**
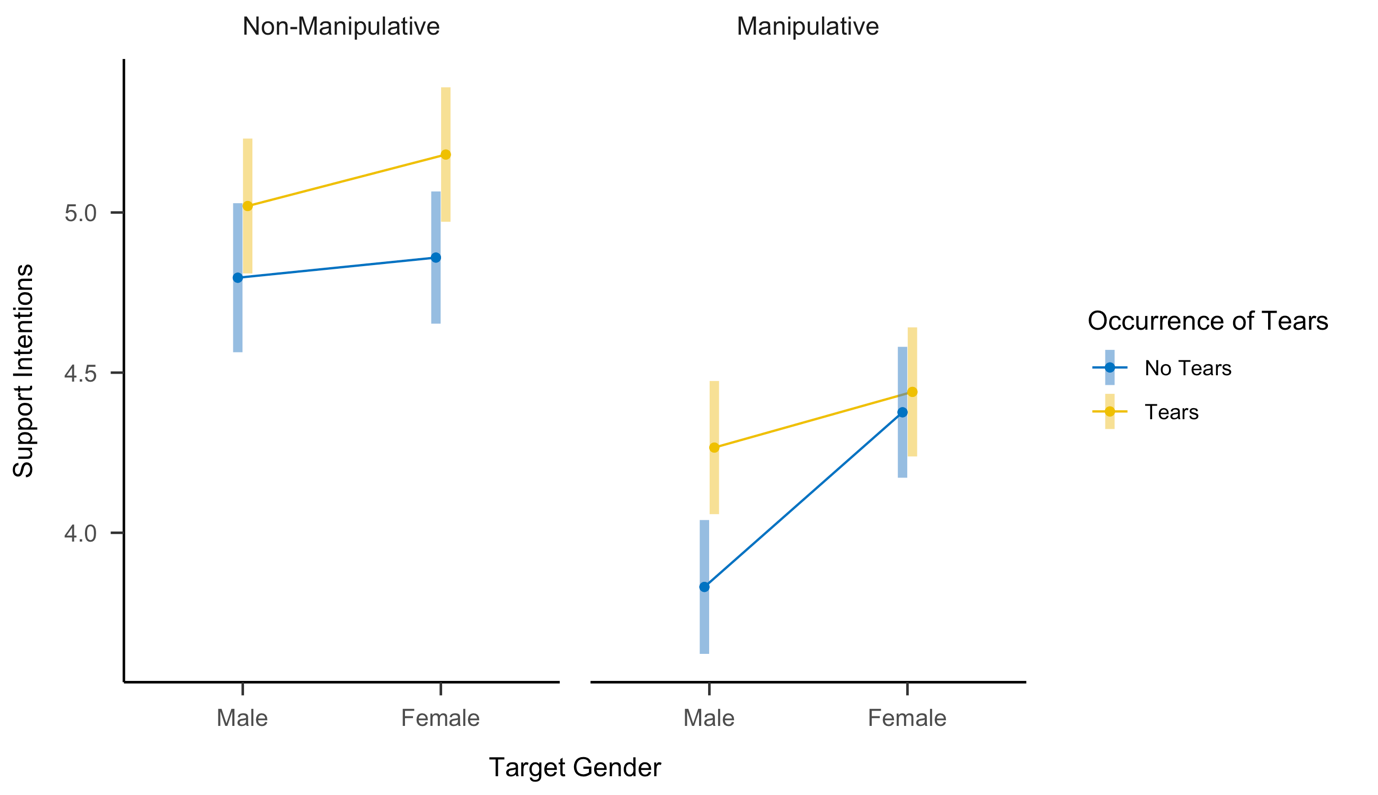
**

**Supplementary Figure S8.** Three-way interaction plot of occurrence of tears, target gender, and situational context (-0.5 = non-manipulative, 0.5 = manipulative) for support intentions. Error bars represent 95% confidence intervals.

**Moderations by Dark Triad (H4)**

**Supplementary Table S28.** Linear regression model with occurrence of tears, situational context, and the Dark triad predictors (mean centered) and their interactions on perceived honesty in Study 2.

|  | **Perceived Honesty** | | | | | |
| --- | --- | --- | --- | --- | --- | --- |
| *Predictors* | *Estimates* | *std. Beta* | *CI* | *standardized CI* | *p* | *std. p* |
| (Intercept) | 4.71 | 0.16 | 4.57 – 4.85 | 0.06 – 0.25 | **<0.001** | **0.002** |
| Occurrence of Tears | 0.08 | 0.06 | -0.11 – 0.28 | -0.08 – 0.20 | 0.397 | 0.396 |
| Situational Context | -0.58 | -0.41 | -0.77 – -0.38 | -0.55 – -0.27 | **<0.001** | **<0.001** |
| Psychopathy | -0.01 | -0.01 | -0.17 – 0.14 | -0.14 – 0.12 | 0.854 | 0.854 |
| Narcissism | 0.07 | 0.06 | -0.08 – 0.22 | -0.07 – 0.19 | 0.378 | 0.378 |
| Machiavellianism | -0.14 | -0.11 | -0.33 – 0.04 | -0.26 – 0.03 | 0.135 | 0.135 |
| OoT x SC | 0.15 | 0.11 | -0.12 – 0.42 | -0.08 – 0.30 | 0.270 | 0.270 |
| OoT ×  Psychopathy | -0.24 | -0.20 | -0.45 – -0.04 | -0.37 – -0.03 | **0.022** | **0.022** |
| SC ×  Psychopathy | -0.19 | -0.16 | -0.40 – 0.02 | -0.33 – 0.02 | 0.079 | 0.079 |
| OoT ×  Machiavellianism | 0.13 | 0.10 | -0.12 – 0.39 | -0.10 – 0.30 | 0.311 | 0.311 |
| SC ×  Machiavellianism | 0.22 | 0.17 | -0.03 – 0.47 | -0.02 – 0.36 | 0.078 | 0.078 |
| OoT ×  Narcissism | 0.03 | 0.03 | -0.17 – 0.24 | -0.15 – 0.21 | 0.771 | 0.771 |
| SC ×  Narcissism | 0.01 | 0.01 | -0.19 – 0.21 | -0.16 – 0.18 | 0.927 | 0.927 |
| OoT x SC x  Psychopathy | 0.16 | 0.13 | -0.13 – 0.45 | -0.10 – 0.37 | 0.268 | 0.268 |
| OoT x SC ×  Machiavellianism | -0.06 | -0.05 | -0.41 – 0.29 | -0.32 – 0.23 | 0.743 | 0.743 |
| OoT x SC ×  Narcissism | -0.10 | -0.09 | -0.38 – 0.17 | -0.33 – 0.15 | 0.468 | 0.468 |
| Observations | 1591 | | | | | |
| R^2^ / R^2^ adjusted | 0.064 / 0.055 | | | | | |

*Note.* OoT = Occurrence of tears, SC = social context. Occurrence of tears (-.5 no tears, .5 tears), social context (-.5 = non-manipulative, .5 = manipulative). All continuous predictors are mean-centered.

**Supplementary Table S29.** Linear regression model of occurrence of tears, situational context, the Dark triad measures, and their interactions on perceived honesty in Study 2.

| *Predictors* | **Perceived Honesty** | | | | | **Honesty** | | | | | **Honesty** | | | | | |
| --- | --- | --- | --- | --- | --- | --- | --- | --- | --- | --- | --- | --- | --- | --- | --- | --- |
|  | *Estimates* | *std. Beta* | *CI* | *standardized CI* | *p* | *Estimates* | *std. Beta* | *CI* | *standardized CI* | *p* | *Estimates* | *std. Beta* | *CI* | *standardized CI* | *p* |  |
| (Intercept) | 4.71 | 0.15 | 4.57 – 4.85 | 0.06 – 0.25 | **<0.001** | 4.71 | 0.16 | 4.57 – 4.86 | 0.06 – 0.26 | **<0.001** | 4.71 | 0.16 | 4.57 – 4.86 | 0.06 – 0.26 | **<0.001** |  |
| Occurrence of Tears | 0.09 | 0.06 | -0.11 – 0.28 | -0.08 – 0.20 | 0.381 | 0.08 | 0.06 | -0.12 – 0.28 | -0.08 – 0.20 | 0.412 | 0.08 | 0.06 | -0.11 – 0.28 | -0.08 – 0.20 | 0.405 |  |
| Situational Context | -0.58 | -0.41 | -0.77 – -0.38 | -0.54 – -0.27 | **<0.001** | -0.57 | -0.40 | -0.76 – -0.37 | -0.54 – -0.26 | **<0.001** | -0.57 | -0.40 | -0.77 – -0.37 | -0.54 – -0.26 | **<0.001** |  |
| OoT x SC | 0.14 | 0.10 | -0.13 – 0.42 | -0.09 – 0.29 | 0.299 | 0.12 | 0.09 | -0.15 – 0.40 | -0.11 – 0.28 | 0.372 | 0.13 | 0.09 | -0.15 – 0.40 | -0.10 – 0.28 | 0.370 |  |
| Psychopathy | -0.07 | -0.06 | -0.19 – 0.05 | -0.16 – 0.04 | 0.270 |  |  |  |  |  |  |  |  |  |  |  |
| OoT ×  Psychopathy | -0.16 | -0.14 | -0.33 – 0.00 | -0.27 – 0.00 | 0.051 |  |  |  |  |  |  |  |  |  |  |  |
| SC ×  Psychopathy | -0.06 | -0.05 | -0.23 – 0.11 | -0.19 – 0.09 | 0.477 |  |  |  |  |  |  |  |  |  |  |  |
| OoT x SC x  Psychopathy | 0.09 | 0.07 | -0.14 – 0.32 | -0.12 – 0.27 | 0.446 |  |  |  |  |  |  |  |  |  |  |  |
| Narcissism |  |  |  |  |  | -0.02 | -0.02 | -0.14 – 0.09 | -0.12 – 0.08 | 0.685 |  |  |  |  |  |  |
| OoT ×  Narcissism |  |  |  |  |  | 0.04 | 0.03 | -0.13 – 0.20 | -0.11 – 0.17 | 0.671 |  |  |  |  |  |  |
| SC ×  Narcissism |  |  |  |  |  | 0.07 | 0.06 | -0.09 – 0.23 | -0.08 – 0.20 | 0.388 |  |  |  |  |  |  |
| OoT × SC ×  Narcissism |  |  |  |  |  | -0.07 | -0.06 | -0.30 – 0.15 | -0.26 – 0.13 | 0.515 |  |  |  |  |  |  |
| Machiavellianism |  |  |  |  |  |  |  |  |  |  | -0.10 | -0.08 | -0.23 – 0.02 | -0.18 – 0.02 | 0.105 |  |
| OoT ×  Machiavellianism |  |  |  |  |  |  |  |  |  |  | -0.01 | -0.00 | -0.18 – 0.17 | -0.14 – 0.13 | 0.947 |  |
| SC ×  Machiavellianism |  |  |  |  |  |  |  |  |  |  | 0.12 | 0.09 | -0.06 – 0.29 | -0.05 – 0.23 | 0.198 |  |
| OoT × SC ×  Machiavellianism |  |  |  |  |  |  |  |  |  |  | -0.03 | -0.02 | -0.28 – 0.22 | -0.22 – 0.17 | 0.811 |  |
| Observations | 1592 | | | | | 1591 | | | | | 1592 | | | | | |
| R^2^ / R^2^ adjusted | 0.056 / 0.052 | | | | | 0.036 / 0.032 | | | | | 0.039 / 0.035 | | | | | |

*Note.* OoT = Occurrence of tears, SC = social context. Occurrence of tears (-.5 no tears, .5 tears), social context (-.5 = non-manipulative, .5 = manipulative). All continuous predictors are mean-centered.

**Moderation by Appropriateness (H5)**

**Supplementary Table S30.** Linear regression with occurrence of tears, target gender, situational context and their interactions on perceived appropriateness in Study 2.

|  | **Perceived Appropriateness** | | | | |
| --- | --- | --- | --- | --- | --- |
| *Predictors* | *Estimates* | *std. Beta* | *CI* | *standardized CI* | *p* |
| (Intercept) | 4.82 | 0.13 | 4.58 – 5.06 | -0.02 – 0.28 | **<0.001** |
| Occurrence of Tears | 0.04 | 0.03 | -0.29 – 0.37 | -0.18 – 0.23 | 0.799 |
| Target Gender | -0.05 | -0.03 | -0.37 – 0.28 | -0.23 – 0.17 | 0.767 |
| Situational Context | -0.68 | -0.43 | -1.01 – -0.35 | -0.63 – -0.22 | **<0.001** |
| OoT × TG | 0.11 | 0.07 | -0.34 – 0.56 | -0.21 – 0.35 | 0.632 |
| OoT x SC | 0.16 | 0.10 | -0.29 – 0.61 | -0.18 – 0.38 | 0.492 |
| TG x SC | 0.48 | 0.30 | 0.03 – 0.93 | 0.02 – 0.58 | **0.035** |
| OoT x TG x SC | -0.34 | -0.21 | -0.96 – 0.29 | -0.60 – 0.18 | 0.291 |
| Observations | 1595 | | | | |
| R^2^ / R^2^ adjusted | 0.026 / 0.022 | | | | |

*Note.* OoT = Occurrence of tears, TG = target gender, SC = social context. Occurrence of tears (-.5 no tears, .5 tears), target gender (-.5 = male, .5 = female), social context (-.5 = non-manipulative, .5 = manipulative).


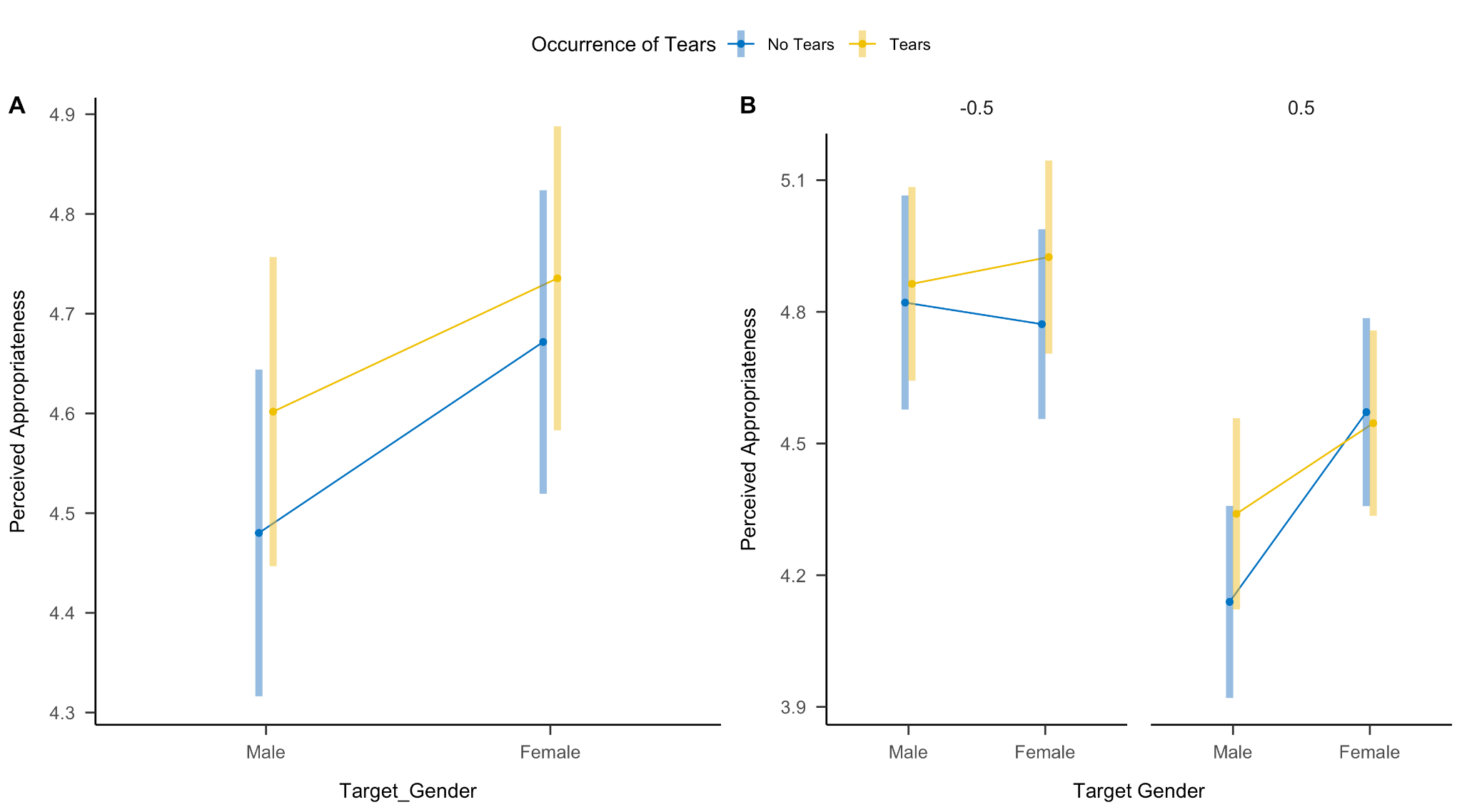


**Supplementary Figure S9.** Two-way interaction between occurrence of tears and target gender (A) and three-way interaction among occurrence of tears, target gender, and situational context (-.5 = non-manipulative, .5 = manipulative) for perceived appropriateness. Error bars represent 95% CIs.
