# Supplementary material for: The honesty behind tears: Situational, individual, and cultural influences on the perception of emotional tears as sincere
Source: PLoS One. 2025 Jul 16;20(7):e0324954. doi: 10.1371/journal.pone.0324954 (PMC12266444; doi:10.1371/journal.pone.0324954)
Supplement: Attachment — Submitted filename: Suggestions for paper revision.docx [file pone.0324954.s006.docx]

Suggestions for paper revision

1. Strengthening the theoretical and literature foundation:

Please provide more theoretical basis for "emotional tears as honesty signals", especially the relevant literature on evolutionary psychology, honest behavior and non-verbal signal theory, to strengthen the theoretical context of this study.

The tension between "crocodile tears" and honest signals deserves further exploration. It is recommended to clearly distinguish the different functions that tears may play in social situations (such as seeking support, manipulation, trust building, etc.).

2. The methodology needs to be explained more clearly and refined:

Please clearly explain what are "posed" and "genuine" tear images, and provide specific examples or verification methods to explain the standards for distinguishing between real and fake tears.

Describe the design details of the manipulative vs. non-manipulative situations and whether the manipulation test method is effective.
